# Supplementary material for: Emergence of a proton exchange-based isomerization and lactonization mechanism in the plant coumarin synthase COSY
Source: Nat Commun. 2023 Feb 3;14:597. doi: 10.1038/s41467-023-36299-1 (PMC9898226; doi:10.1038/s41467-023-36299-1)
Supplement: Supplementary file 1 — Supplementary Information [file 41467_2023_36299_MOESM1_ESM.pdf]

Supplementary information for

Emergence of a proton exchange-based isomerization and lactonization mechanism in the plant coumarin synthase COSY

Kim et al.

*Corresponding author email: wengj@wi.mit.edu

Supplementary Table 1 | Data collection and structure refinement statistics. Data were collected from one crystal per condition and the statistics for the highest resolution shell are shown in parentheses.

Structures	***At*COSY (Apo) PDB ID: 8DQO**	***At*COSY (Scopoletin) PDB ID: 8DQP**	***At*COSY (Umbelliferone) PDB ID: 8DQQ**	***At*COSY (CoA) PDB ID: 8DQR**
**Data Collection**				
Space group	$P2_12_12_1$	$P2_12_12_1$	$P2_12_12_1$	$P2_12_12_1$
Unit cell a, b, c (Å)	57.411, 57.903, 270.395	58.459, 58.349, 273.281	57.907, 58.771, 273.611	58.514, 88.886, 96.901
Ɑ, B, γ (º)	90, 90, 90	90, 90, 90	90, 90, 90	90, 90, 90
R_{merge}	1.361e-17 (1.178e-17)	0.04009 (0.7324)	0.0714 (0.7117)	0.03387 (0.7622)
R_{pim}	1.361e-17 (1.178e-17)	0.04009 (0.7324)	0.0714 (0.7117)	0.03387 (0.7622)
$CC_{1/2}$	1 (1)	0.999 (0.474)	0.991 (0.441)	0.999 (0.425)
I/σ((I)	13.42 (3.53)	10.64 (0.89)	9.40 (1.00)	12.69 (0.89)
Completeness (%)	98.65 (89.64)	97.46 (96.88)	97.43 (92.20)	97.02 (90.69)
**Refinement**				
Resolution range (Å)	48.72 - 1.9 (1.968 - 1.9)	57.17 - 2.48 (2.569 - 2.48)	136.8 - 2.33 (2.413 - 2.33)	50.09 - 2.26 (2.341 - 2.26)
Reflections used in refinement	71349 (6349)	33351 (3261)	40000 (3722)	23599 (2172)
R_{work} / R_{free} (%)	19.6 / 23.0 (23.8 / 25.9)	17.3 / 28.1 (29.2 / 37.7)	25.0 / 30.9 (30.9 / 35.3)	18.8 / 28.4 (38.4 / 39.3)
**Number of atoms**				
Number of non-hydrogen atoms	7232	6718	6862	3586
macromolecules	6835	6555	6734	3388
ligands	46	41	18	55
solvent	351	122	110	143
Protein residues	873	835	862	433
Ramachandran allowed (%)	1.97	4.27	5.19	2.36
Ramachandran outliers (%)	0.12	1.22	0.59	0.71
**R.M.S. deviations**				
Bond lengths (Å)	0.012	0.024	0.003	0.013
Bond angles (º)	1.48	2.35	0.69	1.68
**$B_{factors}$ (Å^2)**				
Average B-factor	34.27	79.56	58.89	54.53
macromolecules	34.16	79.71	58.86	54.36
ligands	41.20	73.51	56.61	57.41
solvent	35.64	73.19	60.93	57.53

Supplementary Table 2 | Statistical analysis of *At*COSY mutant assays. Unpaired, two-tailed t-test was performed using Prism (v9.0)[1] for each mutant vs. WT.

Mutants	p-value	t	df	Significantly different (P < 0.05)?
H161A	0.4433	0.8498	4	no
H161Q	0.0798	2.335	4	no
W371H	0.8868	0.1516	4	no
W371A	0.0058	5.363	4	yes
W371V	0.0110	4.473	4	yes
W371M	0.0066	5.188	4	yes
Y42F	0.0466	2.846	4	yes
F40T	0.3080	1.167	4	no
C164A	0.5104	0.7217	4	no
F40T/Y42S	0.9275	0.09688	4	no
Y42F/H161A	0.0028	6.556	4	yes
G166A	0.1281	1.914	4	no
G166L	0.0107	4.517	4	yes
Y373A	0.9228	0.1052	3	no
Y373F	0.6200	0.5367	4	no
L374A	0.0052	5.540	4	yes
‘Loop-swap’	0.0083	4.854	4	yes

Supplementary Table 3 | Tabulated energetics for the intermediates of the proposed mechanism. Energies are reported in kcal/mol. Reactants are labeled as R and products are labeled as P. Separate geometry optimizations were performed with each functional.

System	B3LYP/6-31G* ΔE (kcal/mol)	wPBEh/6-31G* ΔE (kcal/mol)
R	0.00	0.00
IM_1	6.56	4.88
IM_2	24.00	23.97
IM_3	27.55	30.50
IM_4	7.54	8.94
IM_5	3.91	1.74
P	-22.82	-20.08

Supplementary Table 4 | Strains and plasmids used in this study.

Strain	Description	Source
BL21 (DE3)	*fhuA2 [lon] ompT gal (λ DE3) [dcm] ΔhsdS*	New England BioLabs

Plasmid	Description	Source
pHis8-4b-His$_8$-*At*COSY	His$_8$-*At*COSY (T7), *lacI*, *lacO*, Kan^R, F1-ori	This study
pHis8-4b-His$_8$-*At*COSY H161A	His$_8$-*At*COSY H161A (T7), *lacI*, *lacO*, Kan^R, F1-ori	This study
pHis8-4b-His$_8$-*At*COSY H161Q	His$_8$-*At*COSY H161Q (T7), *lacI*, *lacO*, Kan^R, F1-ori	This study
pHis8-4b-His$_8$-*At*COSY W371H	His$_8$-*At*COSY W371H (T7), *lacI*, *lacO*, Kan^R, F1-ori	This study
pHis8-4b-His$_8$-*At*COSY W371A	His$_8$-*At*COSY W371A (T7), *lacI*, *lacO*, Kan^R, F1-ori	This study
pHis8-4b-His$_8$-*At*COSY W371V	His$_8$-*At*COSY W371V (T7), *lacI*, *lacO*, Kan^R, F1-ori	This study
pHis8-4b-His$_8$-*At*COSY W371M	His$_8$-*At*COSY W371M (T7), *lacI*, *lacO*, Kan^R, F1-ori	This study
pHis8-4b-His$_8$-*At*COSY Y42F	His$_8$-*At*COSY Y42F (T7), *lacI*, *lacO*, Kan^R, F1-ori	This study
pHis8-4b-His$_8$-*At*COSY F40T	His$_8$-*At*COSY F40T (T7), *lacI*, *lacO*, Kan^R, F1-ori	This study
pHis8-4b-His$_8$-*At*COSY C164A	His$_8$-*At*COSY C164A (T7), *lacI*, *lacO*, Kan^R, F1-ori	This study
pHis8-4b-His$_8$-*At*COSY F40T/Y42S	His$_8$-*At*COSY F40T/Y42S (T7), *lacI*, *lacO*, Kan^R, F1-ori	This study
pHis8-4b-His$_8$-*At*COSY Y42F/H161A	His$_8$-*At*COSY Y42F/H161A (T7), *lacI*, *lacO*, Kan^R, F1-ori	This study
pHis8-4b-His$_8$-*At*COSY C308S	His$_8$-*At*COSY C308S (T7), *lacI*, *lacO*, Kan^R, F1-ori	This study
pHis8-4b-His$_8$-*At*COSY C308A	His$_8$-*At*COSY C308A (T7), *lacI*, *lacO*, Kan^R, F1-ori	This study
pHis8-4b-His$_8$-*At*COSY G166A	His$_8$-*At*COSY G166A (T7), *lacI*, *lacO*, Kan^R, F1-ori	This study
pHis8-4b-His$_8$-*At*COSY G166L	His$_8$-*At*COSY G166L (T7), *lacI*, *lacO*, Kan^R, F1-ori	This study
pHis8-4b-His$_8$-*At*COSY Y373A	His$_8$-*At*COSY Y373A (T7), *lacI*, *lacO*, Kan^R, F1-ori	This study
pHis8-4b-His$_8$-*At*COSY Y373F	His$_8$-*At*COSY Y373F (T7), *lacI*, *lacO*, Kan^R, F1-ori	This study
pHis8-4b-His$_8$-*At*COSY L374A	His$_8$-*At*COSY L374A (T7), *lacI*, *lacO*, Kan^R, F1-ori	This study
pHis8-4b-His$_8$-*At*COSY-HCT-loop-swap	His$_8$-*At*COSY-HCT-loop-swap (T7), *lacI*, *lacO*, Kan^R, F1-ori	This study
pHis8-4b-His$_8$-*At*4CL1	His$_8$-*At*4CL1 (T7), *lacI*, *lacO*, Kan^R, F1-ori	This study
pHis8-4b-His$_8$-*Gm*COSY	His$_8$-*Gm*COSY (T7), *lacI*, *lacO*, Kan^R, F1-ori	This study
pHis8-4b-His$_8$-*St*COSY	His$_8$-*St*COSY (T7), *lacI*, *lacO*, Kan^R, F1-ori	This study
pHis8-4b-His$_8$-*Sb*COSY	His$_8$-*Sb*COSY (T7), *lacI*, *lacO*, Kan^R, F1-ori	This study

Supplementary Table 5 | Oligonucleotide sequences reported in this study.

**Name**	**Sequence (5′ - 3′)**
His$_8$-*At*COSY-F	GAAAACTTGTACTTCCAGGCCCATGGCATGGCGACACTTGAAATTACCGATATAGCC
His$_8$-*At*COSY-R	CGGGCTTTGTTAGCAGCCGGATCGCCATGGTCAAGACAGGGCAAATTCACCTTTGTGG
His$_8$-*At*COSY H161A-F (QuikChange)	TCGCAGATCGCAGCATGAATCGATGCACCTAGAACCC
His$_8$-*At*COSY H161A-R (QuikChange)	GGGTTCTAGGTGCATCGATTCATGCTGCGATCTGCGA
His$_8$-*At*COSY H161Q-F (QuikChange)	ATCGCAGATCGCCTGATGAATCGATGCACCTAGAAC
His$_8$-*At*COSY H161Q-R (QuikChange)	GTTCTAGGTGCATCGATTCATCAGGCGATCTGCGAT
His$_8$-*At*COSY W371H-F (QuikChange)	GGAATGCCCCAAGTATCGATGGTCCGTGAATCCGGTCAC
His$_8$-*At*COSY W371H-R (QuikChange)	GTGACCGGATTCACGGACCATCGATACTTGGGGCATTCC
His$_8$-*At*COSY W371A-F (QuikChange)	GCCCCAAGTATCGCGCGTCCGTGAATCCGG
His$_8$-*At*COSY W371A-R (QuikChange)	CCGGATTCACGGACGCGCGATACTTGGGGC
His$_8$-*At*COSY W371V-F (QuikChange)	ATGCCCCAAGTATCGCACGTCCGTGAATCCGGTC
His$_8$-*At*COSY W371V-R (QuikChange)	GACCGGATTCACGGACGTGCGATACTTGGGGCAT
His$_8$-*At*COSY W371M-F (QuikChange)	ATGCCCCAAGTATCGCATGTCCGTGAATCCGGTC
His$_8$-*At*COSY W371M-R (QuikChange)	GACCGGATTCACGGACATGCGATACTTGGGGCAT
His$_8$-*At*COSY Y42F-F (QuikChange)	GACGCGGAGGAAACGGAAGCTGACGTG
His$_8$-*At*COSY Y42F-R (QuikChange)	CACGTCAGCTTCCGTTTCCTCCGCGTC
His$_8$-*At*COSY F40T-F (QuikChange)	CGCGGAGGTAACGGGTGCTGACGTGGAGGT
His$_8$-*At*COSY F40T-R (QuikChange)	ACCTCCACGTCAGCACCCGTTACCTCCGCG
His$_8$-*At*COSY C164A-F (QuikChange)	TCGCACCTAACCCATCGGCGATCGCATGATGAATCG
His$_8$-*At*COSY C164A-R (QuikChange)	CGATTCATCATGCGATCGCCGATGGGTTAGGTGCGA
His$_8$-*At*COSY F40T/Y42S-F (QuikChange)	GGAGTAGACGCGGAGGCTACGGGTGCTGACGTGGAGGTTG
His$_8$-*At*COSY F40T/Y42S-R (QuikChange)	CAACCTCCACGTCAGCACCCGTAGCCTCCGCGTCTACTCC
His$_8$-*At*COSY Y42F/H161A-F (QuikChange)	AGCTTCCGTTTTCTCCGCGT
His$_8$-*At*COSY Y42F/H161A-R (QuikChange)	ACGCGGAGAAAACGGAAGCT
His$_8$-*At*COSY C308S-F (QuikChange)	GCTACTGGGGAAATGGATCAGTGCCAATGTATGCTCAGAT
His$_8$-*At*COSY C308S-R (QuikChange)	ATCTGAGCATACATTGGCACTGATCCATTTCCCCAGTAGC
His$_8$-*At*COSY C308A-F (QuikChange)	GCTACTGGGGAAATGGAGCAGTGCCAATGTATGCTCAGAT
His$_8$-*At*COSY C308A-R (QuikChange)	ATCTGAGCATACATTGGCACTGCTCCATTTCCCCAGTAGC
His$_8$-*At*COSY G166A-F (QuikChange)	GACTCGCACCTAACGCATCGCAGATCGCG
His$_8$-*At*COSY G166A-R (QuikChange)	CGCGATCTGCGATGCGTTAGGTGCGAGTC
His$_8$-*At*COSY G166L-F (QuikChange)	GACTCGCACCTAATAGATCGCAGATCGCG
His$_8$-*At*COSY G166L-R (QuikChange)	CGCGATCTGCGATCTATTAGGTGCGAGTC
His$_8$-*At*COSY Y373A-F (QuikChange)	CGTGGAATGCCCCAAGGCTCGCCAGTCCGTGAAT
His$_8$-*At*COSY Y373A-R (QuikChange)	ATTCACGGACTGGCGAGCCTTGGGGCATTCCACG
His$_8$-*At*COSY Y373F-F (QuikChange)	TGGAATGCCCCAAGAATCGCCAGTCCGTG
His$_8$-*At*COSY Y373F-R (QuikChange)	CACGGACTGGCGATTCTTGGGGCATTCCA
His$_8$-*At*COSY L374A-F (QuikChange)	CGTGGAATGCCCCGCGTATCGCCAGTCCGTG
His$_8$-*At*COSY L374A-R (QuikChange)	CACGGACTGGCGATACGCGGGGCATTCCACG
His$_8$-*At*4CL1-F	ATGGCGCCACAAGAACAAGCAGT
His$_8$-*At*4CL1-R	TCACAATCCATTTGCTAGTTTTGCCC
His$_8$-*Gm*COSY-F	CCAGGCCCATGGCATGCAAAGGATCAAAACCTCAGAAC
His$_8$-*Gm*COSY-R	AGCCGGATCGCCATGGCTATATGTGGCTCAGCCACTGC
His$_8$-*St*COSY-F	CCAGGCCCATGGCATGGAAGTAACTATCGGCGAAAC
His$_8$-*St*COSY-R	CGGATCGCCATGGTCAAGCAAGTCCATGCTCCAACTTTTCC

**Name**	**Sequence (5′ - 3′)**
His_8-*Sb*COSY-F	CCAGGCCCATGGCATGGAGCCACAGCAGCTGCGACTACG
His_8-*Sb*COSY-R	GCAGCCGGATCGCCATGGTCAAAGTTTTCCCGCTGAACACTGC
His_8-*At*COSY-HCT-loop-swap-F	CCAGGCCCATGGCATGGCGACACTTGAAATTACCG
His_8-*At*COSY-HCT-loop-swap-R	GCAGCCGGATCGCCATGGTCAAGACAGGGCAAATTCACC

Supplementary Table 6 | Synthetic gene sequences reported in this study.

Name	Sequence (5′ - 3′)
*Gm*COSY	ATGCAAAGGATCAAAACCTCAGAACGCACTTTGATCTTCCCTTCCCACCCTCCTTTTCTCCAAGACCACCCC TTCCCTCTCTCCCACCTCGACACTGATCCCAACCTCCACCTTACCTTCCGCTACCTCCGCGCGTACACCTC AACAACAACAACAACCTCCCTCGACCCCTTCCACGTCATCTCCTCCTCCCTCTCCCACGCCCTCCCCCACT TCTACCCCCTCACCGCCACCCTCCGCCGCCAACAAACCTCCCCCCACCGCCTCCAACTCTGGTGCGTCG CCGGCCAGGGCATCCCCCTCATCCGCGCCACCGCGGACTTCACCCTCGAGTCCGTGAACTTCCTCGACA ACCCGGCCTCGAGCTTCTTGGAGCAGTTAGTGCCCGACCCGGGACCCGAGGAGGGGATGGAGCACCCG TGCATGCTCCAGGTGACGGTGTTCGCGTGCGGGGGATTCACCCTCGGCGCGGCGATGCACCACGCGCTC TGCGACGGCATGGGCGGGACGCTGTTCTTCAATGCGGTGGCGGAGCTGGCGCGTGGGGCGACCCGGAT AACGTTGGACCCGGTTTGGGACCGTGCGAGGTTGCTGGGTCCCAGGGACCCGCCCCTGGTGGATTCGC CGTTGATTGGGGAGTTTCTGCGTTTGGAGAAGGGAGTTTTGCCGTACCAACAGAGTGTTGGTGGGGTCGC GAGAGAGTGCTTTCACGTGAAGGATGAGTGCTTGGACAATTTCAAGAGGACCTTGTTGGAGCAATCTGGGT TGAACTTCACCGTTTTTGAGGCTCTTGGTGCCTACATCTGGAGGGCTAAGGTCAGGGCCTCGGGAATCCAG GCTGATGAAAAGGTGAAGTTTGCATACTCAATTAACATACGGAGACTGGTAAAGCCACCACTGCCTGGTGGC TATTGGGGTAATGGTTGTGTGCCAATGTACGTACAACTTAGTGCCAAAGATTTGATAGAGAAACCCGTTTGCG AAACCGCAGAGCTAATAAAAAAGAGTAAAAGCAATGTCACTGATGAGTATGTTAAGTCCTACATCGATTATCAG GAGCTGCATTTTGCTGATGGAATCACTGCGGGAAAAGAGGTTAGTGGGTTCACGGATTGGAGGCACTTGGG CCATTCAACTGTGGACTTTGGGTGGGGTGGCCCAGTTACTGTTTTGCCCCTTGGAAGGAACTTACTTGGGA GTGTTGAGCCTTGCTTTTTTTTGCCTTATTCAACAGCCACTTCAGAGAAGAAAGAGGGGTTCAAGGTTTTGG TGACTTTGAGAGAGGCTGCGTTGCCTGCTTTCAGAGAAGACATGAAAGTGTTTTGCAATAGCCAAGAGCAG TGGCTGAGCCACATATAG
*St*COSY	ATGGAAGTAACTATCGGCGAAACTAACACAATCTACCCTTCTAAGCTACCTTTCACTGAAAACCATGTCCTTC CTCTCTCCCATATTGACACCGATCGGAACCTCAATTTCACTTTCCGATACCTCCGTGTCTACGTCAACGACG ACACGACACAACAAAAACCAGACCCATATGAAGCTCTCACCTCCTCCCTCTCCGCCGCTCTCGTCCACTAC TACCAATTCGCCGGCTCTCTCCGCCGTCGTCCGTCGGACAACCGTCTAGAGCTTCACTGTCAAGTAGGGAA TGGCGTTCCCGTAATTCTGTCCACGGTGGATTGTACCTTGGCTTCTATTAACTACCTGGATGATCCAGATTATA ACTTCGCTGAGAAGCTGGTACCCGACCCGAGAGATGAGGAGGTGTTGACCCGACCCTTGATTTTGCAACT GAACCGTTTTAAGTGTGGTGGGTGGGTTTTCGGAACGGCGGTTCATCACGCGATGTGTGATGGAATGGGAT CGACGCTGTTTTTTCATGCTATGGCGGAGATTGCTCGTGGGGAGAACGGGATGAAGATTGAGCCGGTGTG GGACCGGTCGAATTTGCTTGGACCGAGGAATCCACCGCGAGTTGAATTTCCGGTTCATGAGTTTCTCAGTT TGGATAGGGATTCGTCTCCATACTTGGAATCAGATAAACCGGCGGTTCGAGAGTGCTTTGAGGTGAAGGATG AGTGGTTGGATAGGCTAAAGGGGTTTCTTCATGAGCAATCCGGTTCAAATTATACAACTTTTGAAGCTTTGGG AGCTTTCATATGGCGAGCAAAGGCAAAGGCTTCTAAAATCTCAGATGATGAGACAGTGAAGTATGCCTATCTA ACCAATATCAGAAGAAGAGTGAAGCCACCATTGCCAGCAGGTTACTGGGGCAATGGGTGCGTGCCAATATA CGTTCAGCTCCTCGCAAAGGACCTCATCAATGAACCTATCTGGAAAGCAGCAGATGCGATAAAGAAGAGCA AGGACATCATCACAGATGAGTATGTTCATTCGTTCATTGATTTCCAGGAGCTGCATTATGATGAAGGGATCACA TCAGGGAATAGAGTGAGTGCATTTACAGATTGGCGGCATGTAGGCCACGAGACGGTTGATTTTGGATGGGG TGGCCCTGTGACTGTCTTTCCTCTGTCTAGACACTTGGTTGGGAGTGTTGAACCTTGTTTTTTCTTGCCTTATT CTTCCGCCACTCAAGGTAAGAAAGATGGTTTCAAAGTTTTGGTCTGTCTGCAACAAGAAGCCATGCCTGTTT TCATGGAAGAGATGGAAAAGTTGGAGCATGGACTTGCTTGA
*Sb*COSY	ATGGAGCCACAGCAGCTGCGACTACGAATTCTGGACACCGTGCGCCTGTCGCCGCCACCCGCCCCGGC GCAGCACGCGGCGGCCCTCCCGCTCTCCGGCCTCGACGCCGACCGCAACGCGCTCCACGTCACCTTCC GCACGCTCCGCTTCTTCCCACCTCCCCCGCCGTCCATCGACCCCCACGCGGTCCTCCCGCCCGCGTTC GAGGCCGCGCTCGGTCTCTTCCCGGCGCTCGCGGGCCGCCTCCGCGTCCGCGACGGCCACGTTGTGG TCGGCGCTGCCGCCGTGCCCGTCGTCCTCGCGGAGTCCGGTCTGTCCGCGGCCGACGTCGACACCGAC TGCCCCGGCTCGGCGCTGCTCGACCACCTCGCGCCGCCGGGGGACGGCGACGCGGATACCCCGGTGC TCGCGCTCCAGGCCACGCGGTTCGCGTGCGGCGGCGTCGCGCTCGGGATGCGGGTCGCGCACGCCCT CTGCGACGGCGCGGGCGCCACCAAGTTCCTCGCCGCCGCGGCGCGGTTCGCGCGGGGGCAGGGGAT GCCGGAGGTGGCGCCTGTGTGGGACCGGCGGGAGCTGCTGGGCCCGAGGCGCCCGCCGCGCGTCGC GACGCTGGTGTTCGACCGCGTCCTCGCGCTCGACGGCGGCGTCGCCCGGTGTGGGCCGTACGGAGCC GTGCGGGAATGGCGCGAACAGCAGCGGCAGCTGACGAGGGAATGCTTCCACGTGAGCAACGCGCGCGT GGAGGCGCTGAGAGCGCGGATCGCCGACGAGGCTGGCGTCAAGCTCACGACCTTCGAGGTTGTCGCAG CGTTCATCTGGCGCGCCAGGGTCAAGGCAAACGGGACCAGCTCCGGCGAGGTGGTGAAGATGGTGTACT CCATGAACATCAGCAAGCTCGTCGACCCGCCGCTCCCCGACGGGTACTGGGGCAACGTGTGCGTCCCTG TCTACGTCACCCTGGCCGCCTGCGACCTCGTCGCCCAGCCACTGGCGGCAACCGCCGCCCTGATAAAGA AGAGCAAGCAGGCCGTGGACGACGAGTACGTGCGATCCTACGTCGACTTCCAGGAGCTGCACCGCCACG ATGGGGTCACGGCGGGAGCCGTGAGTGCGTTCACGGACTGGCGGCGGCTCGGCCATGGGGAGGTGGA CTTCGGGTGGGGCGGCCCCGACTCCGTGCTGCCGCTCTCGTGGAGGATCCTCGGGAGCACGGAGCCCT GCTTCCTGCTGCCCTACGGCGCGGGGGATGAGCGGCGGCGGGGAGGGTTCAAGGTGTTCATCGCGCTG CAGCGCACGGCGCTGGCTGGTTTCAGAGAGGAAATGCAGGAGCTGCTGTTGCAACCGAAGCAGTGTTCA GCGGGAAAACTTTGA

Name	Sequence (5′ - 3′)
*At*COSY-HCT-loop-swap	ATGGCGACACTTGAAATTACCGATATAGCCCTGGTTCAACCTTCTCACCAACCACTCTCCAACGACCAAACT<br>CTCTCTCTTTCCCATCTCGACAATGATAACAACCTCCACGTCAGCTTCCGTTACCTCCGCGTCTACTCCTCCT<br>CCTCTTCCACCGTCGCCGGAGAAAGCCCCTCTGCCGTCGTATCCGCCTCTCTTGCCACCGCTCTCGTTCA<br>CTACTACCCTCTTGCTGGCTCTCTCCGTCGCTCTGCCTCCGATAACCGATTTGAACTACTCTGCTCCGCTGG<br>TCAAAGCGTGCCTTTAGTCAACGCTACAGTGAACTGTACGCTTGAGTCAGTCGGGTATTTGGATGGACCCGA<br>TCCAGGTTTCGTCGAGAGATTGGTACCGGATCCGACCCGGGAGGAAGGAATGGTCAATCCTTGTATCCTCC<br>AGGTCACTATGTTTCAGTGTGGTGGTTGGGTTCTAGGTGCATCGATTCATCATGCGATCTGCGATGGGTTAGG<br>TGCGAGTCTGTTCTTCAACGCTATGGCGGAATTAGCTCGCGGAGCGACAAAGATTTCGATCGAACCGGTTT<br>GGGACAGAGAACGTTTACTTGGTCCAAGGGAGAAGCCTTGGGTTGGAGCTCCAGTTCGTGACTTCTTGAG<br>CCTGGATAAGGACTTTGATCCATATGGACAAGCCATTGGAGACGTCAAAAGAGACTGCTTCTTTGTGACCGA<br>CGATTCTTTGGATCAATTGAAGGCTCAATTACTCGAGAAATCGGGTCTGAATTTCACCACATTCGAAGCTCTT<br>GGTGCTTACATTTGGCGTGCAAAGGTAAGAGCTGCAAAGACTGAGGAAAAGGAGAATGTGAAATTTGTGTAT<br>TCGATAAATATAAGGAGATTGATGAATCCACCTTTGCCTAAAGGCTACTGGGGAAATGGATGTGTGCCAATGT<br>ATGCTCAGATCAAAGCTGGAGAACTCATTGAGCAACCAATCTGGAAAACTGCAGAGCTCATAAAACAGAGCA<br>AATCCAATACGAGTGATGAATATGTACGCTCCTTTATCGACTTCCAAGAGCTGCATCACAAAGATGGAATCAAT<br>GCCGGTACAGGAGTGACCGGATTCACGGACTGGCGATACTTGGGGCATTCCACGATTGATTTTGGATGGG<br>GAGGACCTGTGACGGTTTTGCCAGGTGGAATTCCATACGAGGGTTTGTCTTTTTTCTTGCCATATTCTACTGA<br>TGCTGCAGCTGGAAGCAAGAAAGACAGTGGGTTTAAGGTTTTGGTAAATCTGCGCGAATCTGCAATGCCTG<br>AGTTTAAAGAGGCCATGGATAAGTTCCACAAAGGTGAATTTGCCCTGTCTTGA

Supplementary Figures

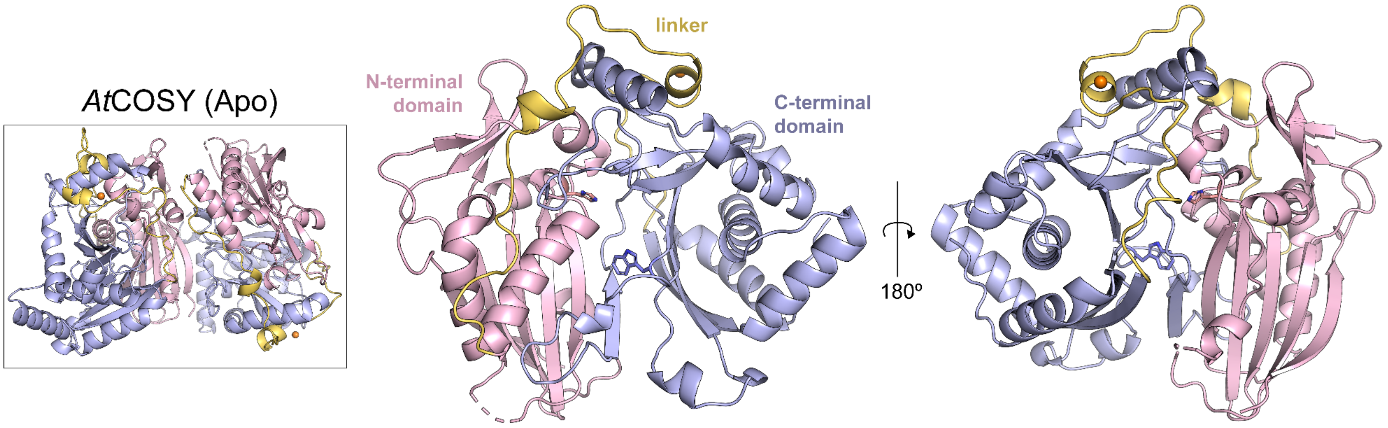

Supplementary Fig. 1 | Apo structure of *At*COSY. Apo structure of *At*COSY at 1.9 Å resolution. The overall fold showcases a pseudo-symmetric N-terminal (residues 1-181; light pink) and C-terminal (residues 230-451; light blue) domains, connected by a linker loop (residues 182-229; yellow). Ca^{2+} ion is displayed as an orange sphere near the linker. The conserved BAHD catalytic residues (His161 and Trp371) are showcased within the active site pocket.

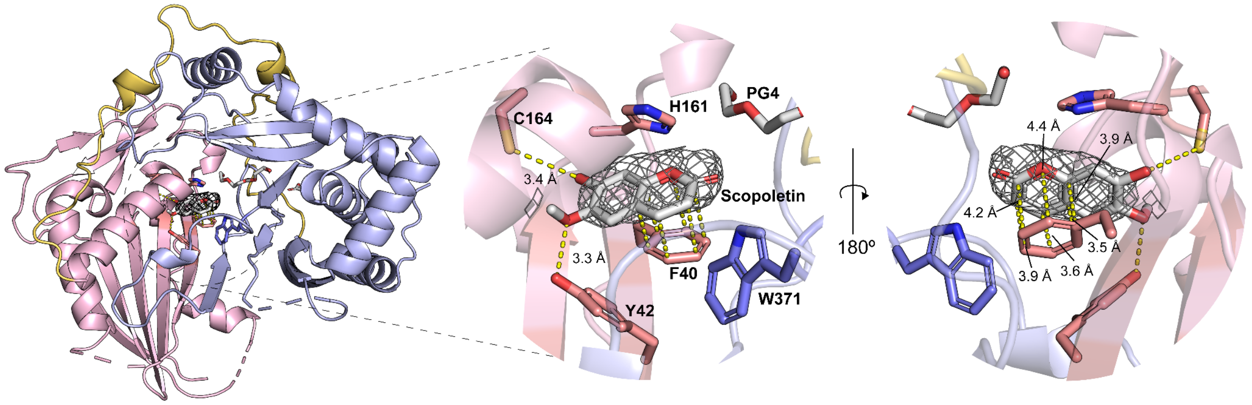

Supplementary Fig. 2 | Active-site residues involved in scopoletin stabilization and catalysis. Scopoletin-bound structure of *At*COSY is displayed in N-terminal (residues 1-181; light pink) and C-terminal (residues 230-451; light blue) domains, connected by a linker loop (residues 182-229; yellow). The conserved BAHD catalytic residues (His161 and Trp371), along with product stabilizing residues (Phe40, Tyr42, and Cys164) are showcased within the active site pocket. The mFo – DFc simulated annealing omit map with electron density map around SCO ligand is contoured at 1.0 σ.

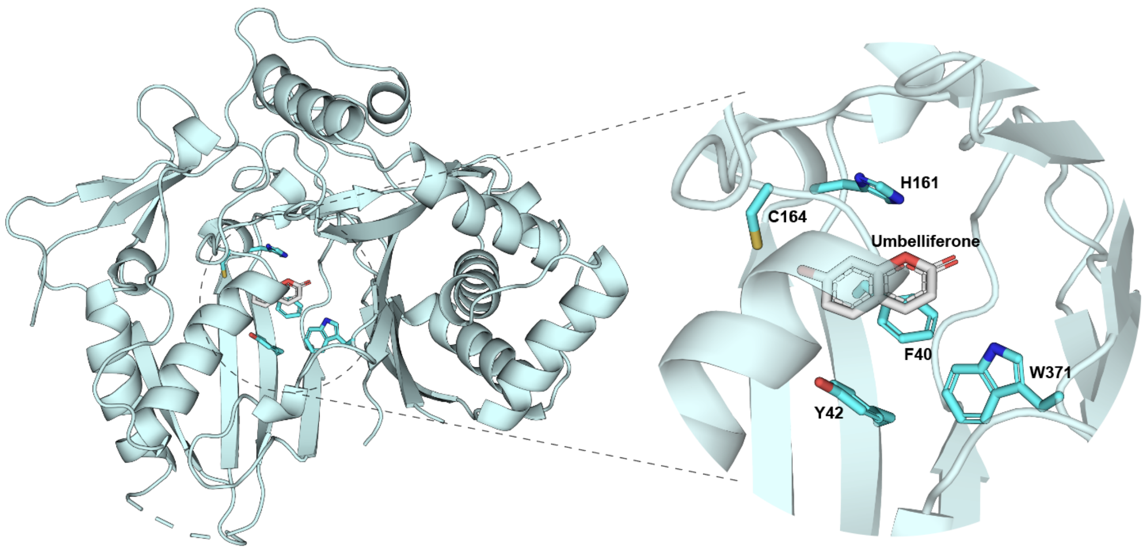

Supplementary Fig. 3 | Structure of *At*COSY co-crystallized with umbelliferone. The conserved BAHD catalytic residues (His161 and Trp371), along with product stabilizing residues (Phe40, Tyr42, and Cys164) are showcased within the active site pocket.

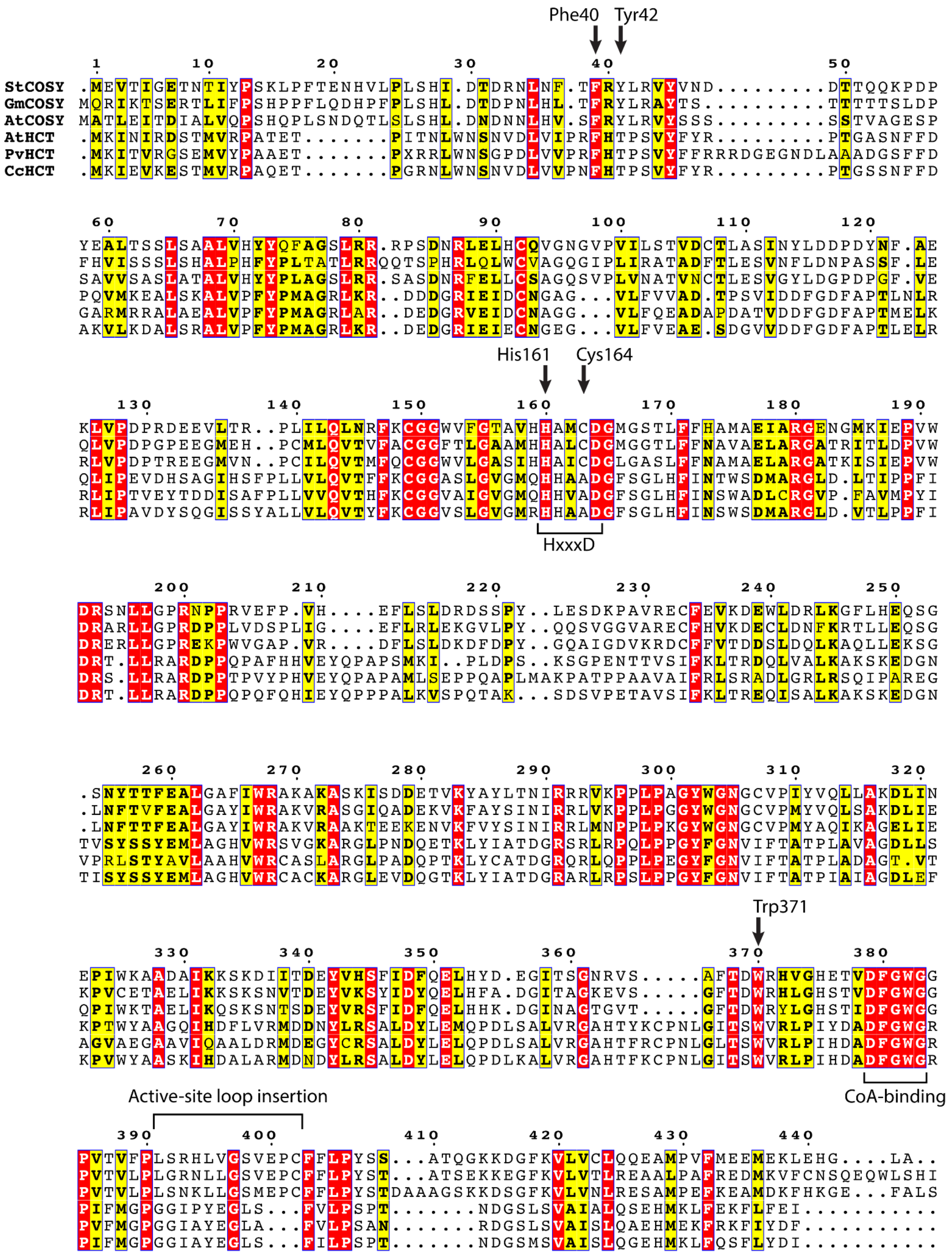

Supplementary Fig. 4 | Sequence alignment of COSY and HCT sequences. COSY homologous sequences were selected from *A. thaliana* (NCBI Accession # NP_174189.1), *S. tuberosum* (NCBI Accession # KAH0704856.1), and *G. max* (NCBI Accession # XP_003541712.1). HCT homologous sequences were selected from *A. thaliana* (NCBI Accession # NP_199704.1), *P. virgatum* (NCBI Accession # AGM90558.1) and *C. canephora* (NCBI Accession # ABO47805.1). Black arrows indicate residues involved in stabilization of scopoletin and canonical residues of BAHD acyltransferases. Conserved sequence motifs in BAHD acyltransferases: HxxxD (catalytic domain) and DFGWG (CoA binding domain) are highlighted, along with the observed active-site loop insertion in COSY. The multiple sequence alignment was built using MUSCLE[2] and the figure was generated using ESPript3.0[3].

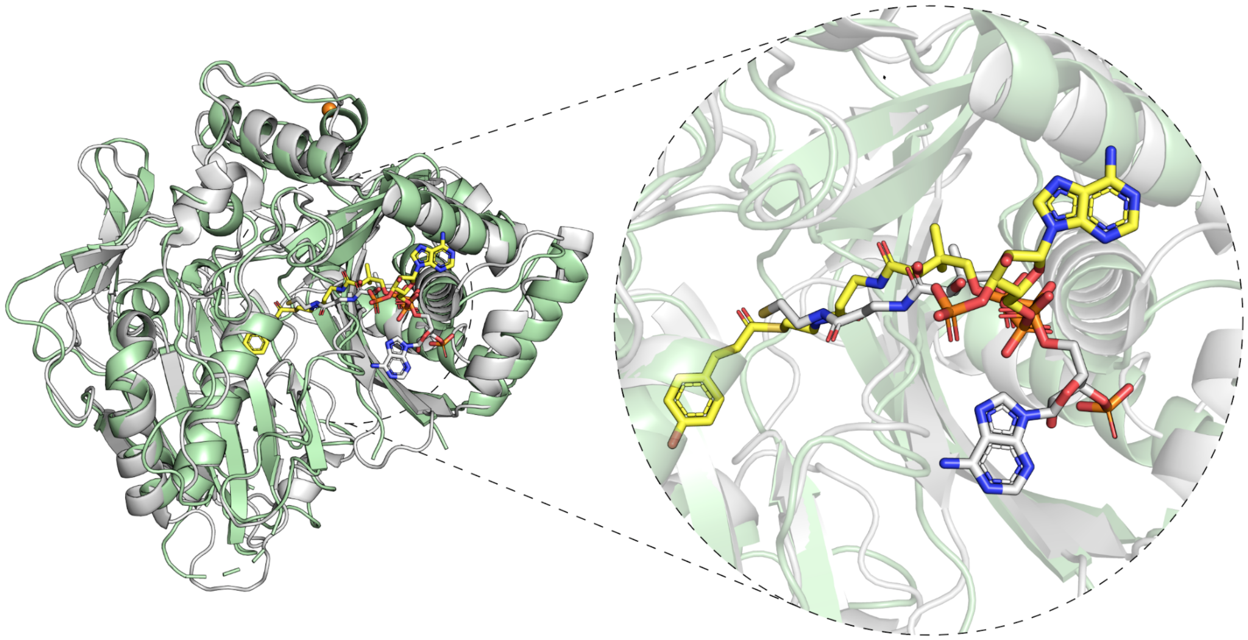

Supplementary Fig. 5 | Structural alignment of *At*COSY-CoA and *At*HCT. Free-CoA bound *At*COSY structure highlighted in green is aligned to *At*HCT highlighted in gray (PDB: 5KJT). CoA from *At*COSY structure is colored in white, whereas p-coumaroyl-CoA from *At*HCT is colored in yellow.

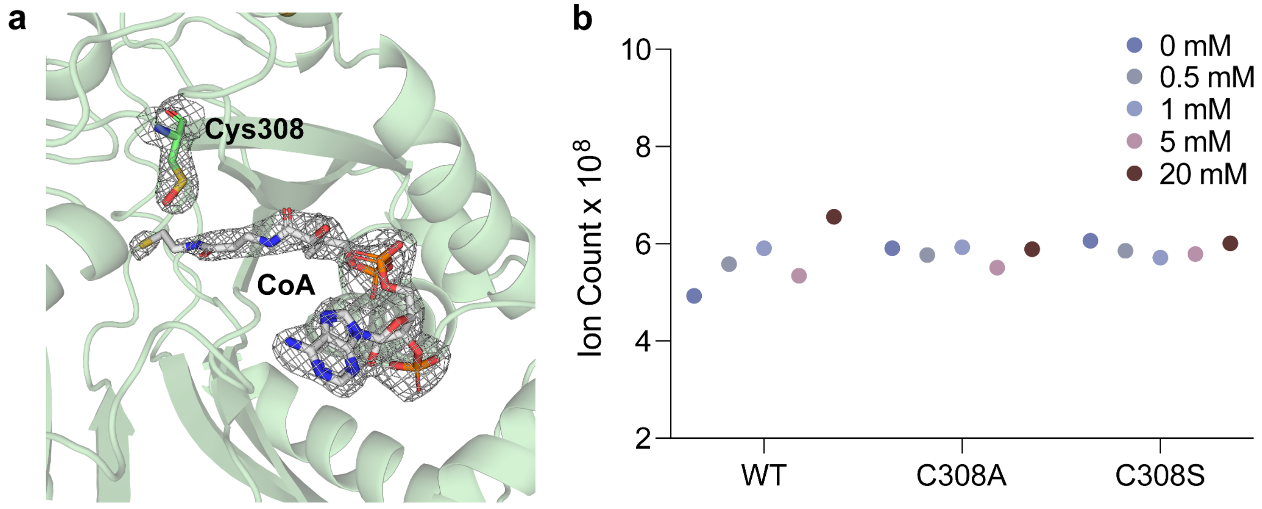

Supplementary Fig. 6 | Observation of Cys380 oxidation in *At*COSY structure and assay for redox-dependency. (**a**) Free-CoA bound *At*COSY structure highlighted in green portrays a single oxidation of Cys308 to sulfenic acid oxidation. |2mFo – DFc| electron density map for Cys308 and CoA (white) is contoured at 1.0 σ. (**b**) Total ion count of scopoletin from LC-MS for WT, C308A, and C308S enzymatic assays in duplicates across varying [H_2O_2]. Data are presented as mean values of duplicate experiments. Relevant quantitative data underlying this figure is provided as a Source Data file.

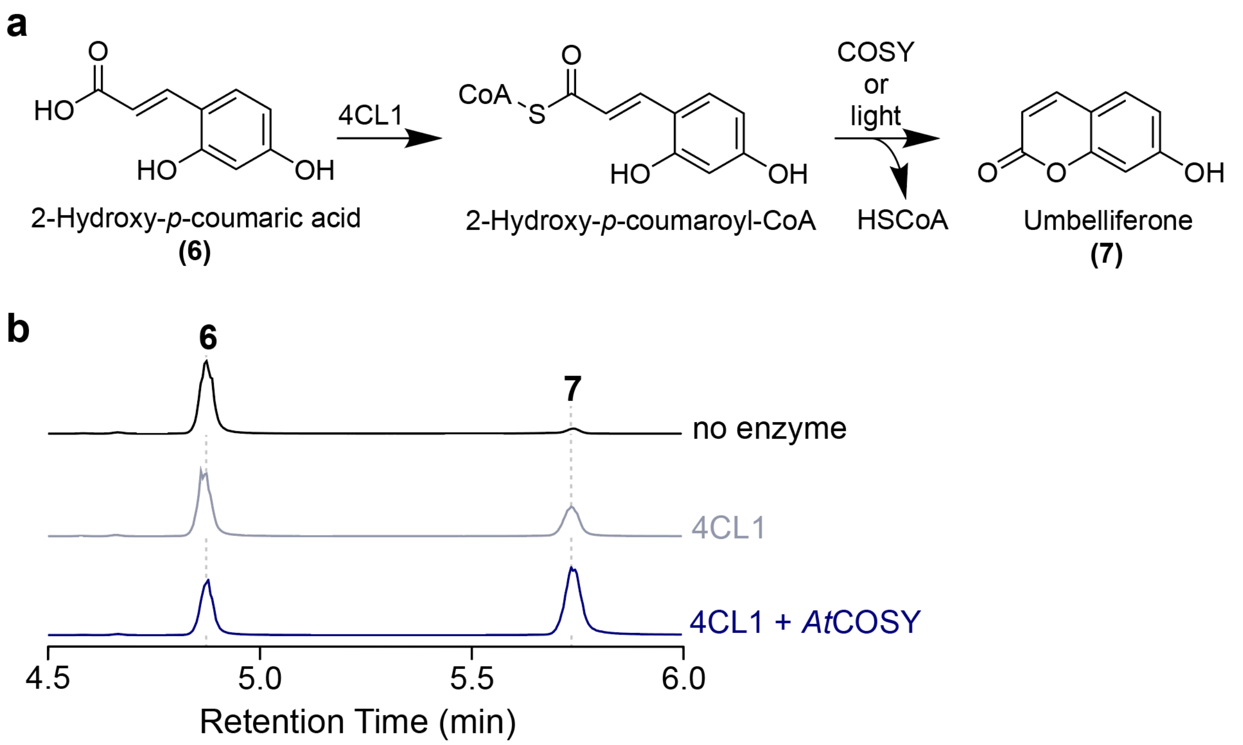

Supplementary Fig. 7 | *In vitro* biochemical assay of *At*COSY for *trans-cis* isomerization and lactonization activity on 2OHpCA. (**a**) Reaction schematic of the conversion of 2-hydroxy-*p*-coumaric acid (2OHpCA) to umbelliferone using 4-coumarate-CoA ligase 1 from *A. thaliana* (*At*4CL1) and coumarin synthase (*At*COSY). (**b**) Combined LC-MS extracted ion chromatograms (XICs) of 135.04512, 179.03494 *m/z*; **6** and 161.02428 *m/z*; **7**. As umbelliferone production is spontaneously catalyzed by light, 4CL1 without *At*COSY sample serves as an experimental control for the coumarin synthase activity of *At*COSY. 4CL1+COSY sample shows complete conversion of 2OHpCA into umbelliferone.

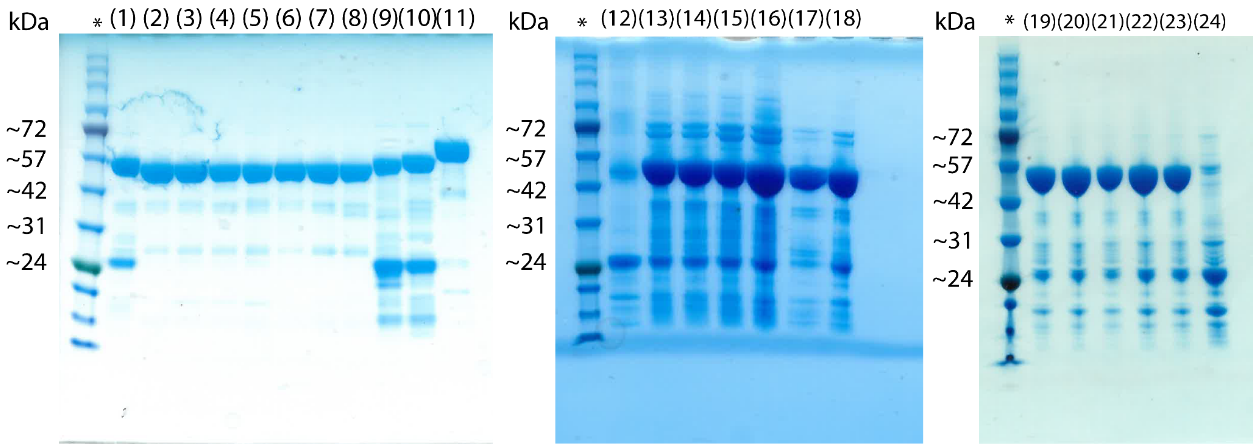

Supplementary Fig. 8 | Purified recombinant proteins assayed in this study. The lanes in the gel correspond to the following: (1) *At*COSY WT, (2) *At*COSY H161A, (3) *At*COSY Y42F, (4) *At*COSY Y42F/H161A, (5) *At*COSY H161Q, (6) *At*COSY F40T, (7) *At*COSY C164A, (8) *At*COSY F40T/Y42S, (9) *Gm*COSY WT, (10) *St*COSY WT, (11) *At*4CL1 WT, (12) *Sb*COSY WT, (13) *At*COSY W371H, (14) *At*COSY W371A, (15) *At*COSY W371V, (16) *At*COSY W371M, (17) *At*COSY C308S, (18) *At*COSY C308A, (19) *At*COSY L374A, (20) *At*COSY Y373A, (21) *At*COSY Y373F, (22) *At*COSY G166A, (23) *At*COSY G166L, and (24) *At*COSY$^{\text{HCT-Loop-swap}}$. (1) and (19)-(24) have N-terminal 8xHis-tags. The asterisk indicates the lane for BlueStain^{TM} protein ladder (GoldBio). The SDS-PAGE experiment was repeated twice independently with similar results.

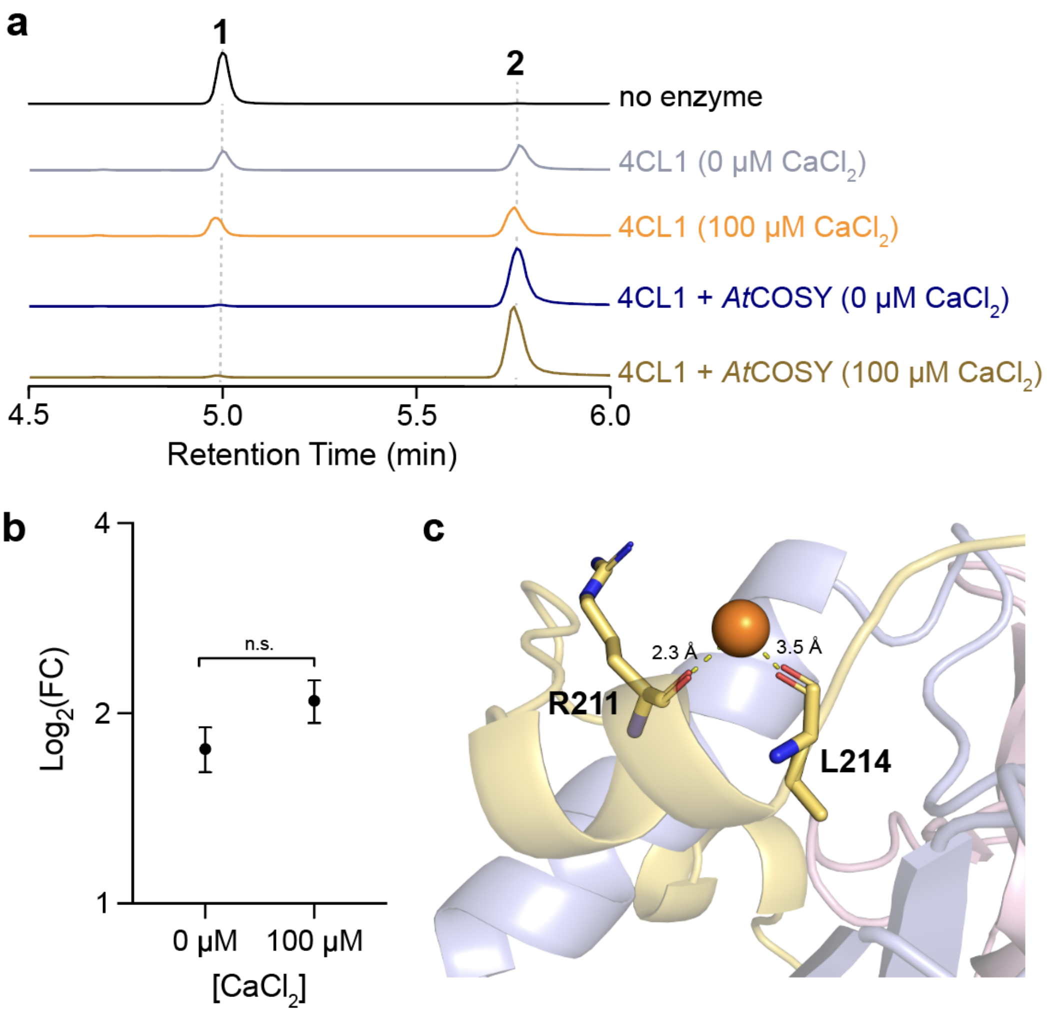

Supplementary Fig. 9 | Observation of Ca^{2+} ion in *At*COSY structures and assay for Ca^{2+} dependency. (**a**) Combined LC-MS extracted ion chromatograms (XICs) of 135.04512, 179.03494 *m/z*; **6** and 161.02428 *m/z*; **7**. As umbelliferone production is spontaneously catalyzed by light, 4CL1 without *At*COSY sample serves as an experimental control for the coumarin synthase activity of *At*COSY. 4CL1+COSY sample shows complete conversion of 2OHpCA into umbelliferone. (**b**) *In vitro* assay for testing Ca^{2+} dependency. Unpaired, two-tailed t-test for statistical comparison of the coumarin synthase activity compared to the respective *At*4CL1 only control. 3 replicates of 0 µM and 100 µM [$CaCl_2$] were added for the enzyme assays. Data are presented as mean values +/- standard error of the mean. Relevant quantitative data underlying this figure is provided as a Source Data file. (**c**) Ca^{2+} ion present in the scopoletin-bound structure. Backbone carbonyls of R211 and L214 coordinate the Ca^{2+} ion.

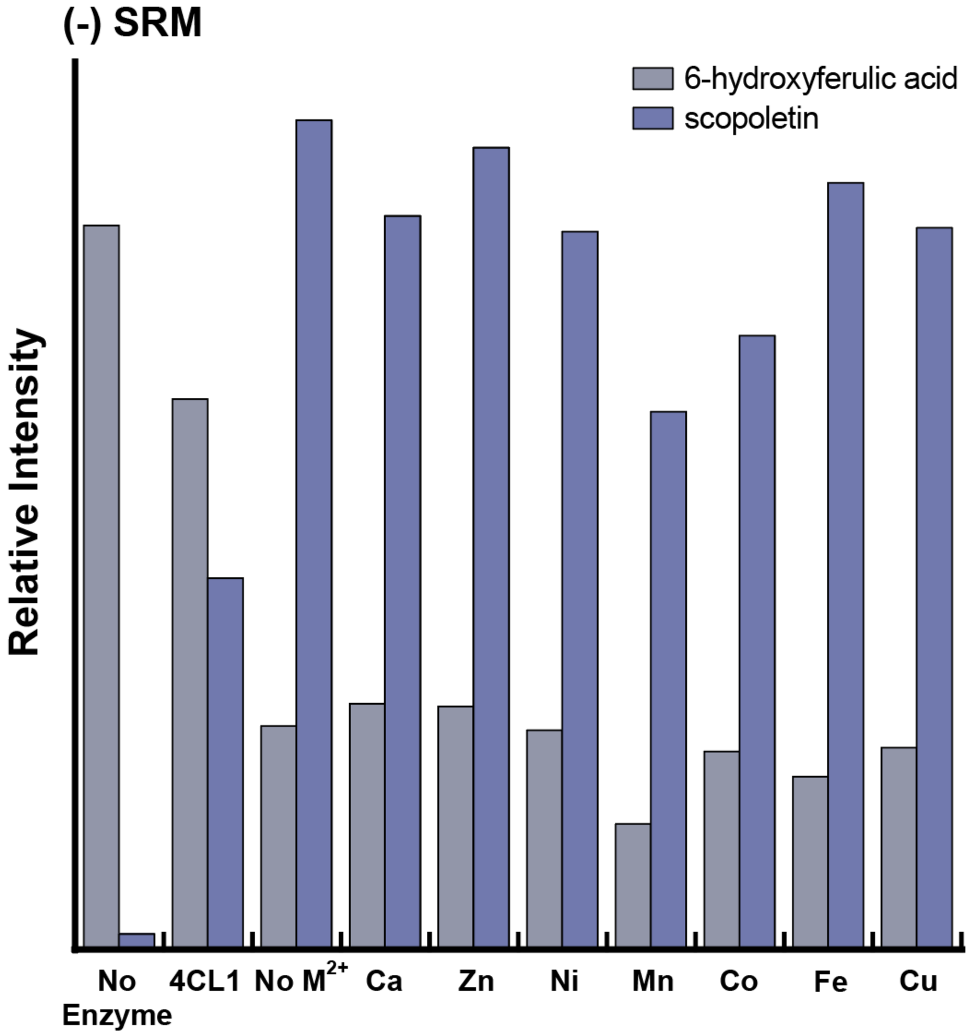

Supplementary Fig. 10 | Assay of divalent cation-dependent coumarin synthase activity in *At*COSY. The enzyme assay was carried out by adding each of the following sources of divalent cations $CaCl_2$, $ZnCl_2$, $NiCl_2$, $MnCl_2$, $CoCl_2$, $FeSO_4$ and $CuCl_2$ at 100 µM each. Single reaction monitoring (SRM) operated in negative ionization mode was used to detect the $[M\text{-}H]^-$ *m/z* values of the expected compounds: 165.1 *m/z* to 150.1 *m/z*; **1** and 191.1 *m/z* to 176.1 *m/z;* **2** both with mass windows of 0.5 *m/z*. The peak area for each sample is reported as relative intensity. This mass spectrometric analysis was performed on a TSQ Quantum Access Max mass spectrometer (Thermo Fisher Scientific).

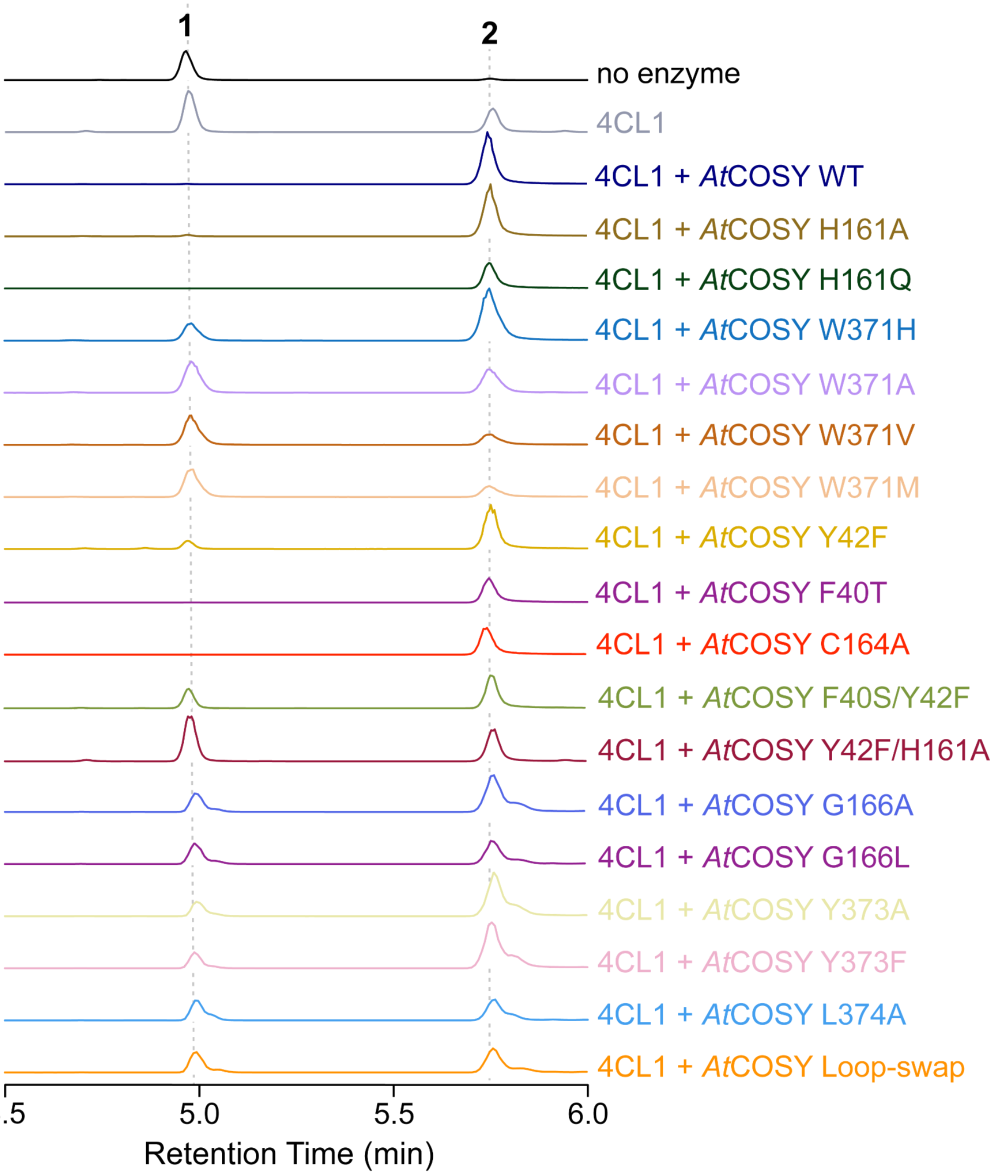

Supplementary Fig. 11 | Qualitative LC-MS analysis of *At*COSY mutant assays. XICs showing the highest conversion of 6OHFA; **1** to scopoletin; **2** for each of the mutant assays are selected and highlighted in various colors corresponding to each mutant (n=3). W371H, W371A, W371V, W371M, G166A, G166L, Y373A, Y373F, L374A, and 'Loop-swap' mutant assays were performed separately from other mutants with proper WT control and scale-adjusted based on the WT assays. Mass windows used for displaying the XICs: 165.05556 m/z and 209.04549 m/z; **1**, and 191.03443 m/z; **2**. There are no side products observed in these assays.

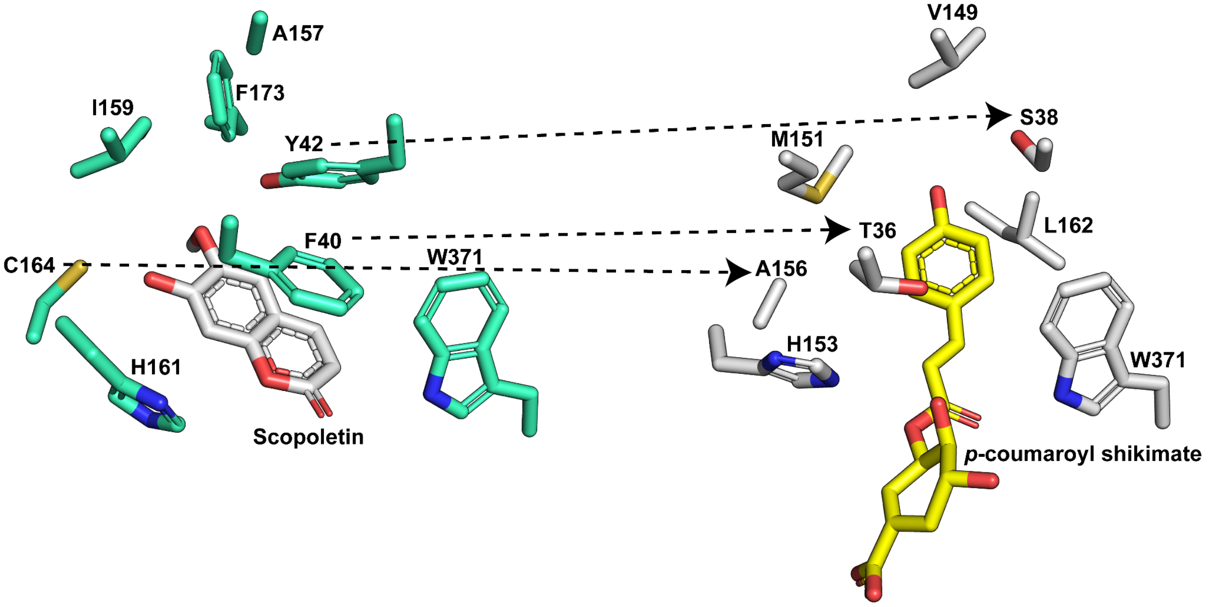

Supplementary Fig. 12 | Structural comparison between *At*COSY and *At*HCT. The active site comparison between *At*COSY and *At*HCT revealed several substitutions at corresponding scopoletin stabilizing residues (F40->T36; Y42->S38; C164->A156).

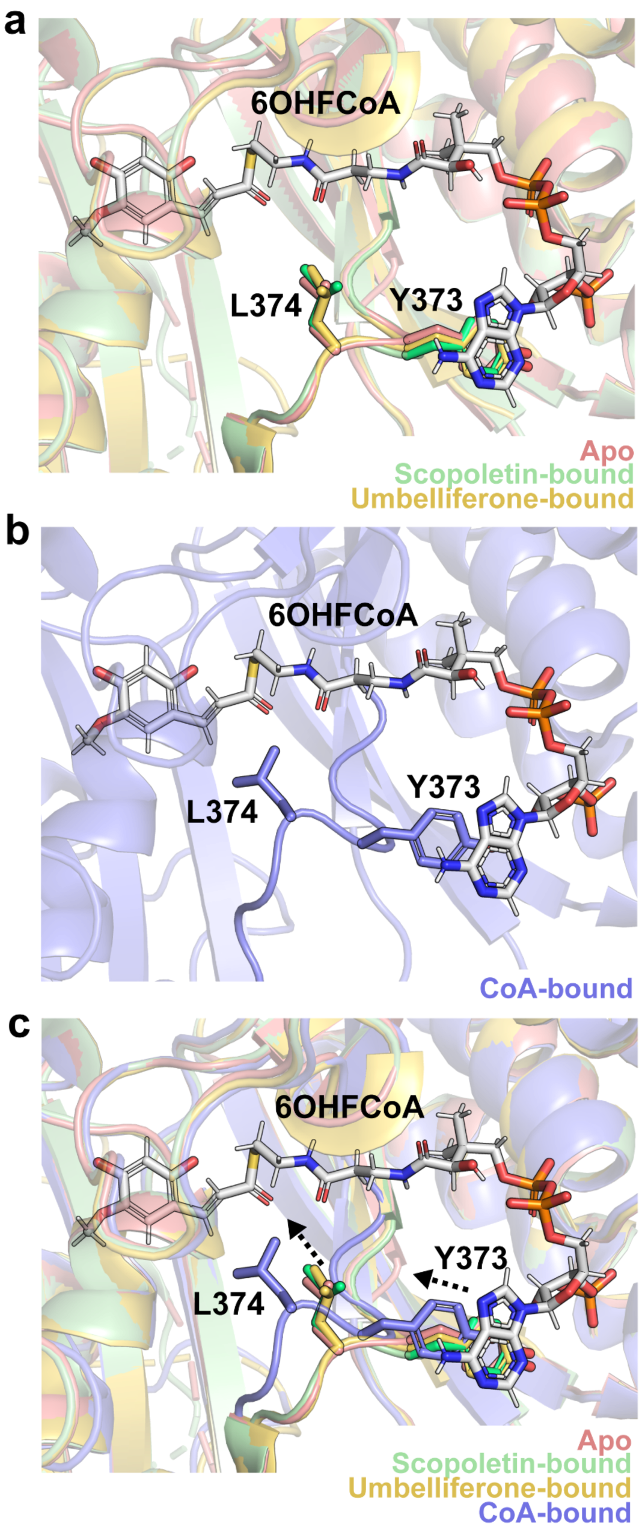

Supplementary Fig. 13 | Structural alignment of active-site loop in obtained *At*COSY structures. The dynamic loop containing Tyr373 and Leu374 is shown relative to *trans*-6OHFCoA in structural alignment of (**a**) apo, scopoletin-bound, and umbelliferone-bound *At*COSY structures; (**b**) CoA-bound *At*COSY structure (**c**) all *At*COSY structures reported in this study. Arrows indicate change in orientation of Tyr373 and Leu374 in CoA-bound *At*COSY structure compared to others in structural alignment.

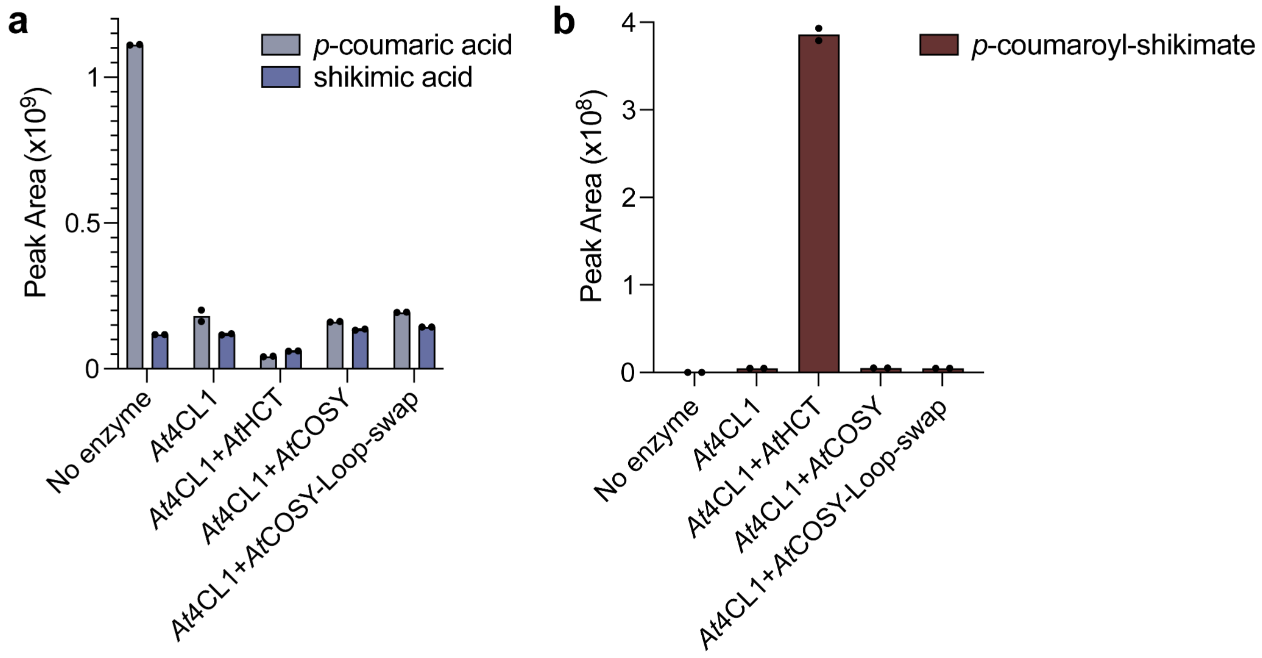

Supplementary Fig. 14 | Assay of *p*-coumaroyl-shikimate producing activity in *At*COSY active-site-loop domain swap assay. (**a**) Total ion count of the LC-HRAM-MS peak area of *p*-coumaric acid ($[M-H]^-$ = 163.0400 *m/z*) and shikimic acid ($[M-H]^-$ = 173.0457 *m/z*) for no enzyme, *At*4CL1-only, *At*4CL1+*At*HCT, *At*4CL1+*At*COSY, and *At*4CL1+*At*COSY-Loop-swap mutant. Duplicate independent experiments were examined over 1 independent experiment (n=2) with data portraying the mean value. (**b**) Total ion count of the LC-HRAM-MS peak area of *p*-coumaroyl-shikimate ($[M-H]^-$ = 319.0823 *m/z*). Duplicate independent experiments were examined over 1 independent experiment (n=2) with data portraying the mean value. Qualitative comparison of the *p*-coumaroyl-shikimate producing activity between *At*4CL1+*At*COSY-Loop-swap mutant and *At*4CL1-only sample indicated no significant difference. Relevant quantitative data underlying this figure is provided as a Source Data file.

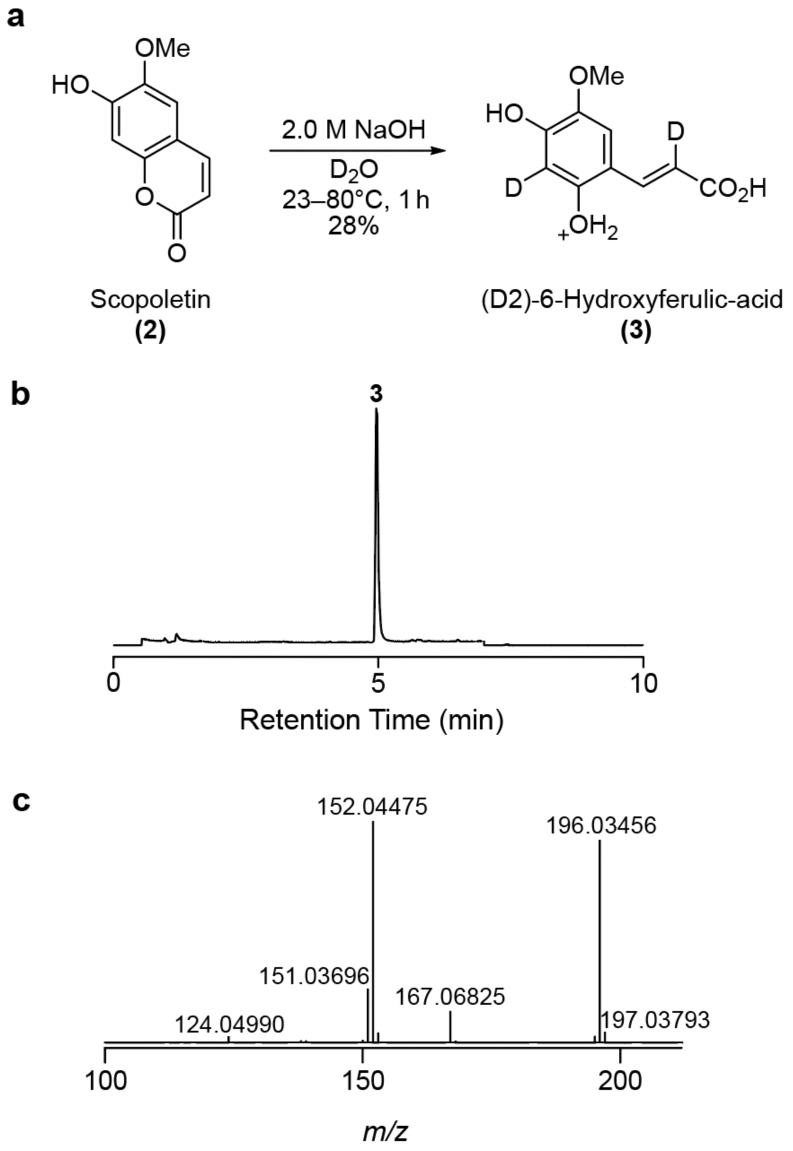

Supplementary Fig. 15 | Chemical synthesis of D2-6OHFA. (**a**) Reaction diagram of saponification reaction in D_2O of scopoletin (**2**) to (D2)-6-hydroxyferulic-acid (**3**). (**b**) TIC of the purified **3** shows no significant side-products from the deuterated-saponification reaction and purification process. (**c**) MS^2 fragmentation spectra of 211.0581 *m/z*; **3**, with the major peaks 196.03456 *m/z* and 152.04475 *m/z*.

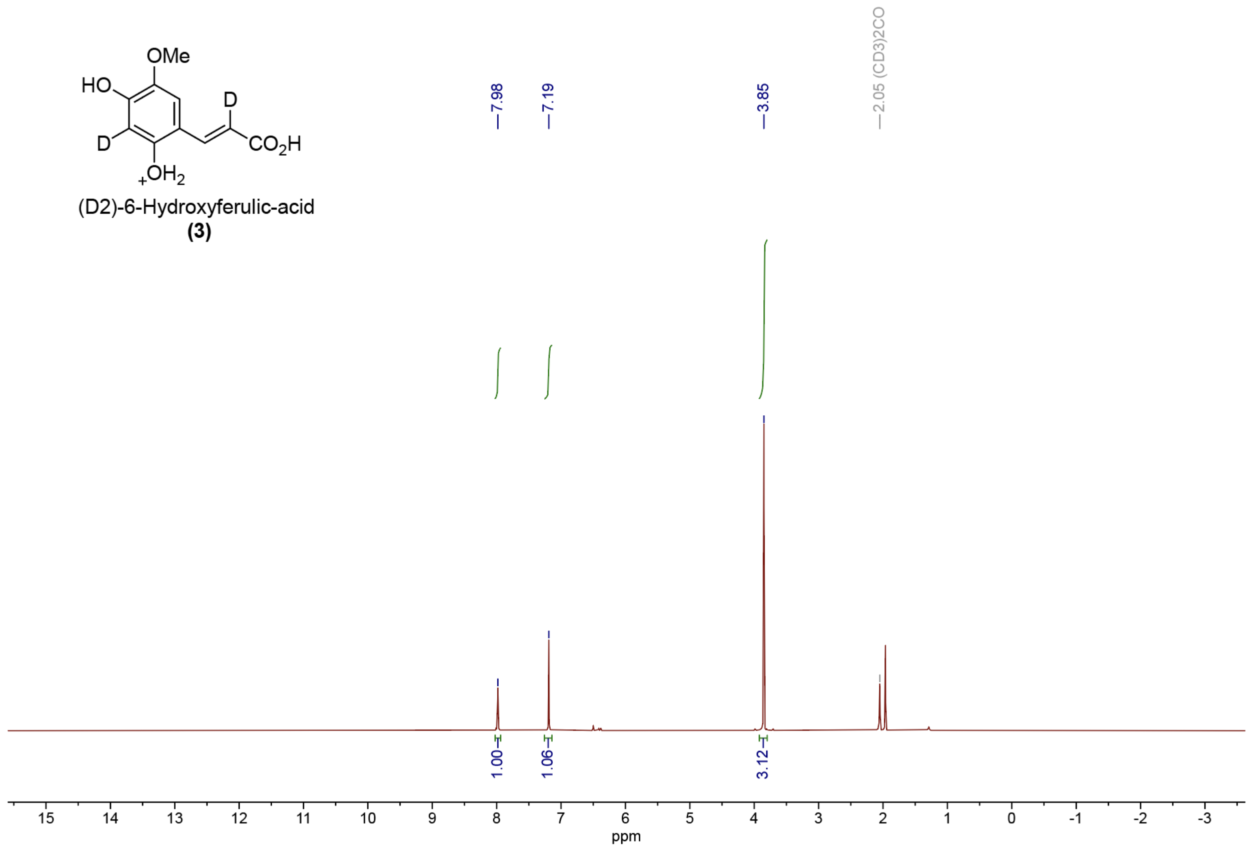

Supplementary Fig. 16 | ^{1}H-NMR spectra of D2-6OHFA. ^{1}H NMR (acetone-d6, 500 MHz) δ 7.98 (s, 1H), 7.19 (s, 1H), 3.85 (s, 3H).

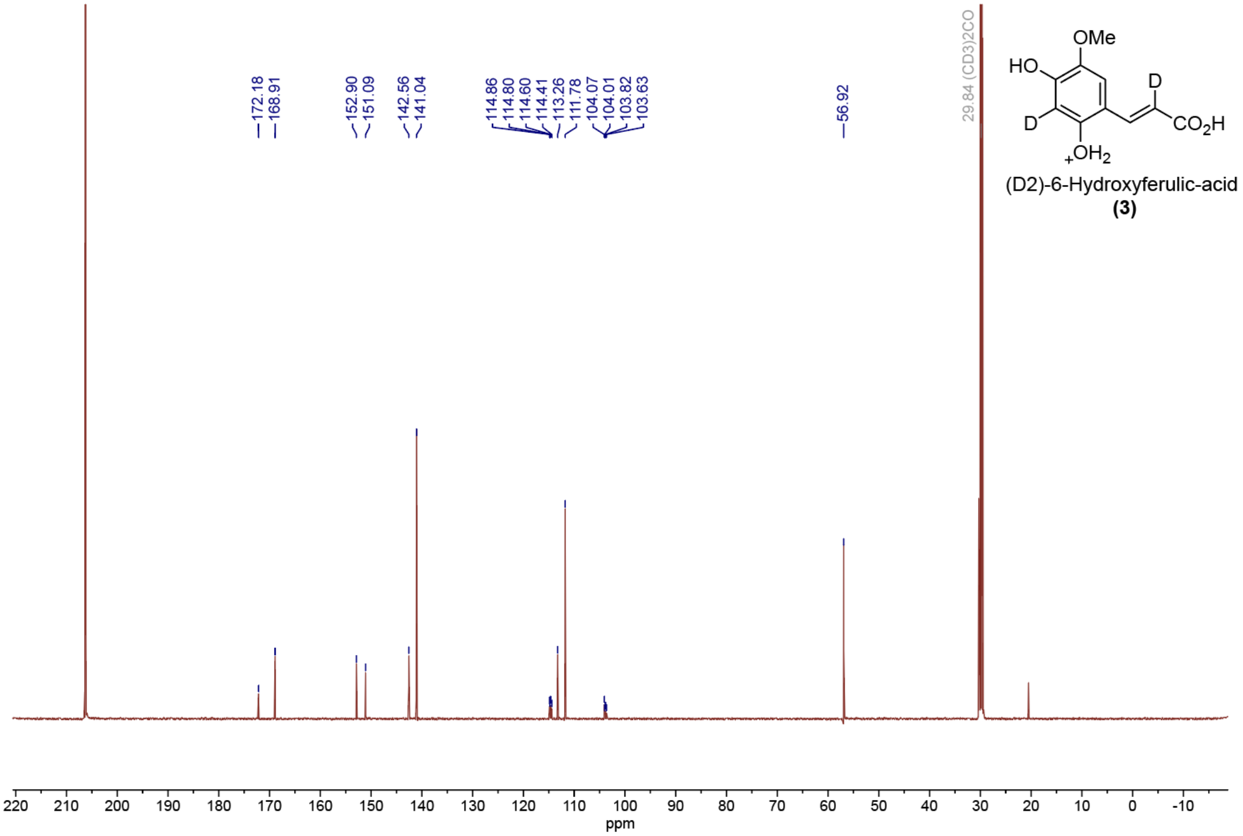

Supplementary Fig. 17 | ^{13}C-NMR spectra of D2-6OHFA. ^{13}C NMR (acetone-d6, 125 MHz) δ 172.2, 168.9, 152.9, 151.1, 142.6, 141.0, 114.9, 114.8, 114.6, 114.4, 113.3, 111.8, 104.1, 104.0, 103.8, 103.6, 56.9.

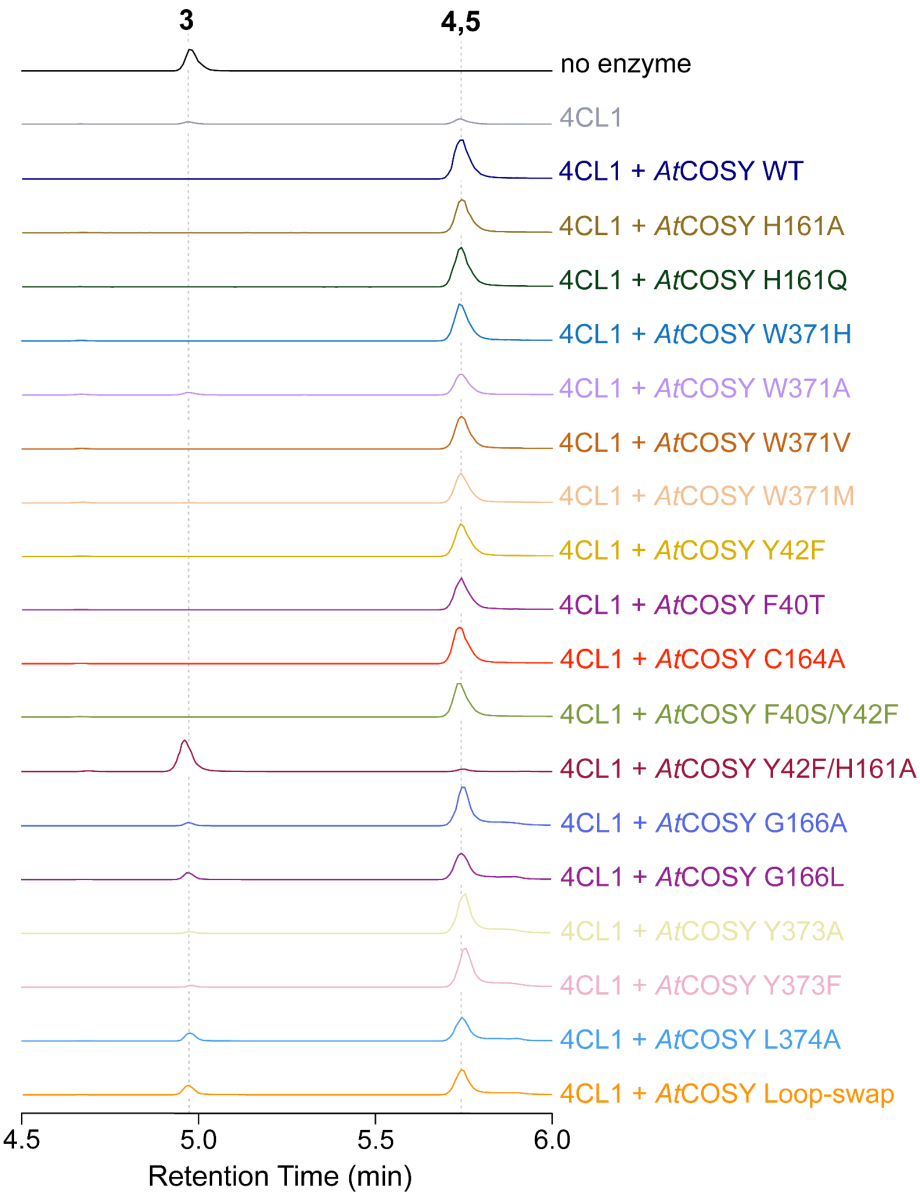

Supplementary Fig. 18 | Qualitative LC-MS analysis of *At*COSY mutant assays against D2-6OHFA. XICs showing the highest conversion of D2-6OHFA (**3**) to the product (**4, 5**) for each of the mutant assays are selected and highlighted in various colors corresponding to each mutant (n=2). G166A, G166L, Y373A, Y373F, L374A, and 'Loop-swap' mutant assays were performed separately from other mutants with proper WT control and scale-adjusted based on the WT assay. Mass windows used for displaying the XICs: **3**, 167.06804 m/z; **4**, 193.04747 m/z; and **5**, 192.04114 m/z. There are no side products observed in these assays.

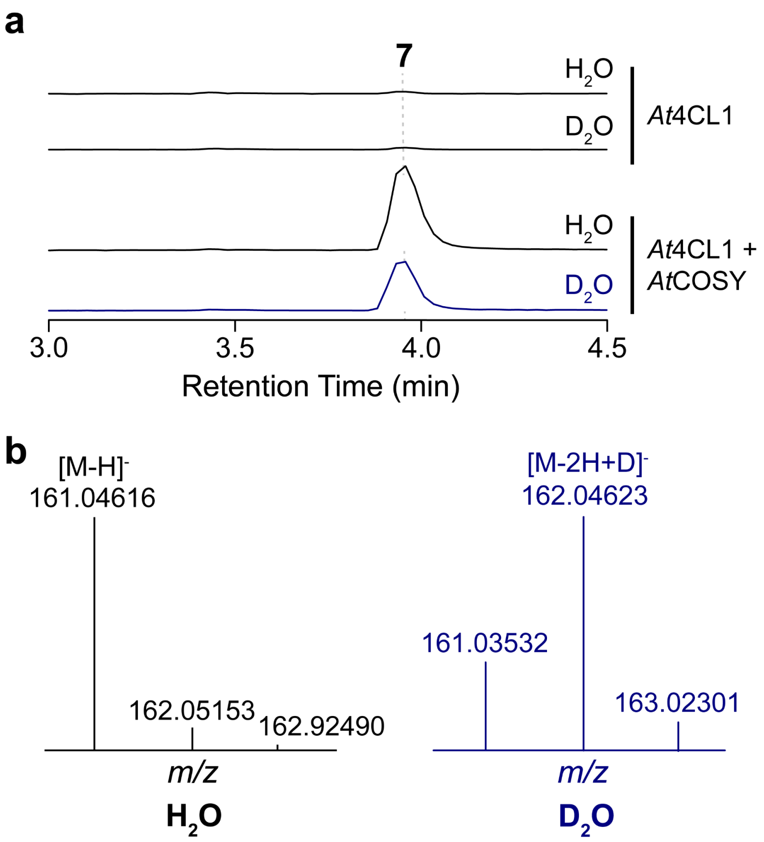

Supplementary Fig. 19 | Enzyme activity of WT *At*COSY in D_2O against 2OHpCACoA. (**a**) Single ion monitoring (SIM) of umbelliferone in enzymatic assay with *At*4CL1 (top two traces) and *At*4CL1+WT *At*COSY (bottom two traces) in 100% H_2O or 66% D_2O. LC-MS trace in blue showcases the umbelliferone peak with H/D-exchange. (**b**) MS^1 spectra of umbelliferone products obtained under SIM mode. WT *At*COSY reaction in 100% H_2O yielded umbelliferone product with 161.04616 *m/z* (black) $[M-H]^-$, whereas in 66% D_2O yielded product with 162.04623 *m/z* (blue) corresponding to the $[M-2H+D]^-$ value of umbelliferone.

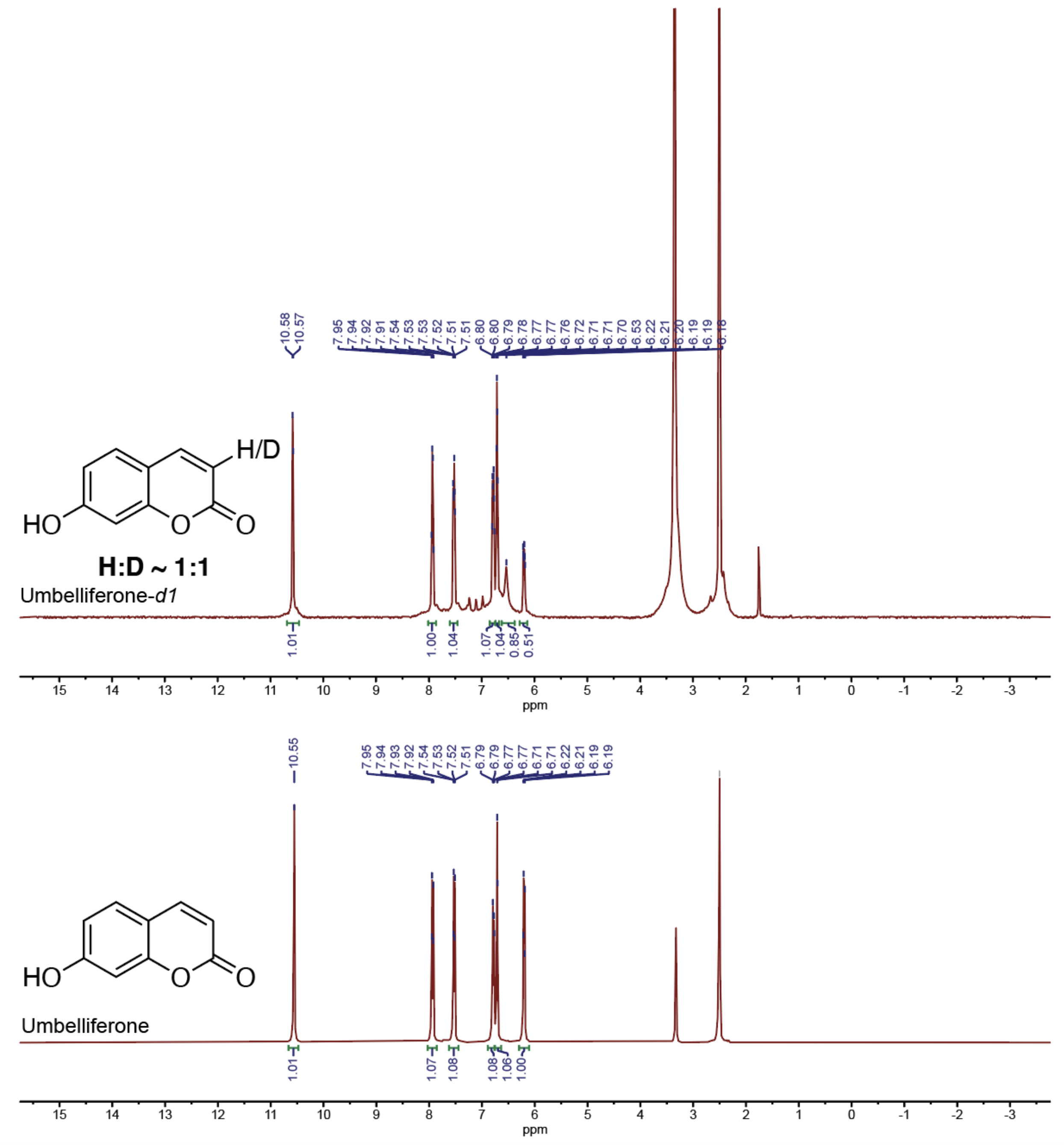

Supplementary Fig. 20 | ^{1}H NMR spectra of umbelliferone-*d1* and umbelliferone. A comparison of umbelliferone-*d1* (top) and umbelliferone (bottom) ^{1}H NMR spectra. The peak at 6.19 ppm corresponding to C2 proton integrates to approximately half in the umbelliferone-*d1* spectra compared to umbelliferone spectra.

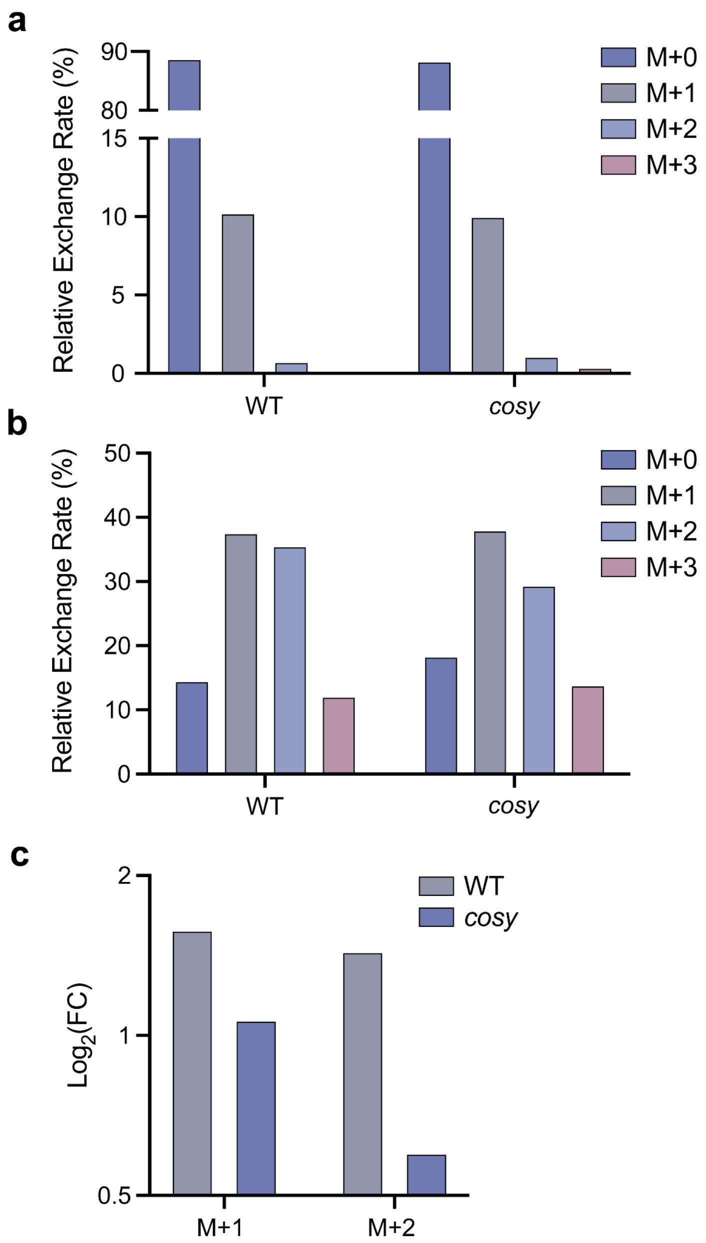

Supplementary Fig. 21 | *In planta* feeding of D_2O for WT and *cosy A. thaliana*. Relative H/D exchange rate for scopoletin (M = 191.03508 m/z) and its corresponding isotopes (M+1 = 192.04123 m/z; M+2 = 193.04744 m/z; M+3 = 194.05373 m/z) observed in (**a**) WT and *cosy* roots grown in H_2O, and (**b**) WT and *cosy* roots grown in 100% D_2O. (**c**) Log_2 fold-change of M+1 and M+2 peaks compared to the corresponding parent scopoletin peak in WT and *cosy* roots grown in 100% D_2O. All assays were performed in 3 technical replicates and small error bars representing the standard error of the mean are not shown at this scale. Relevant quantitative data underlying this figure is provided as a Source Data file.

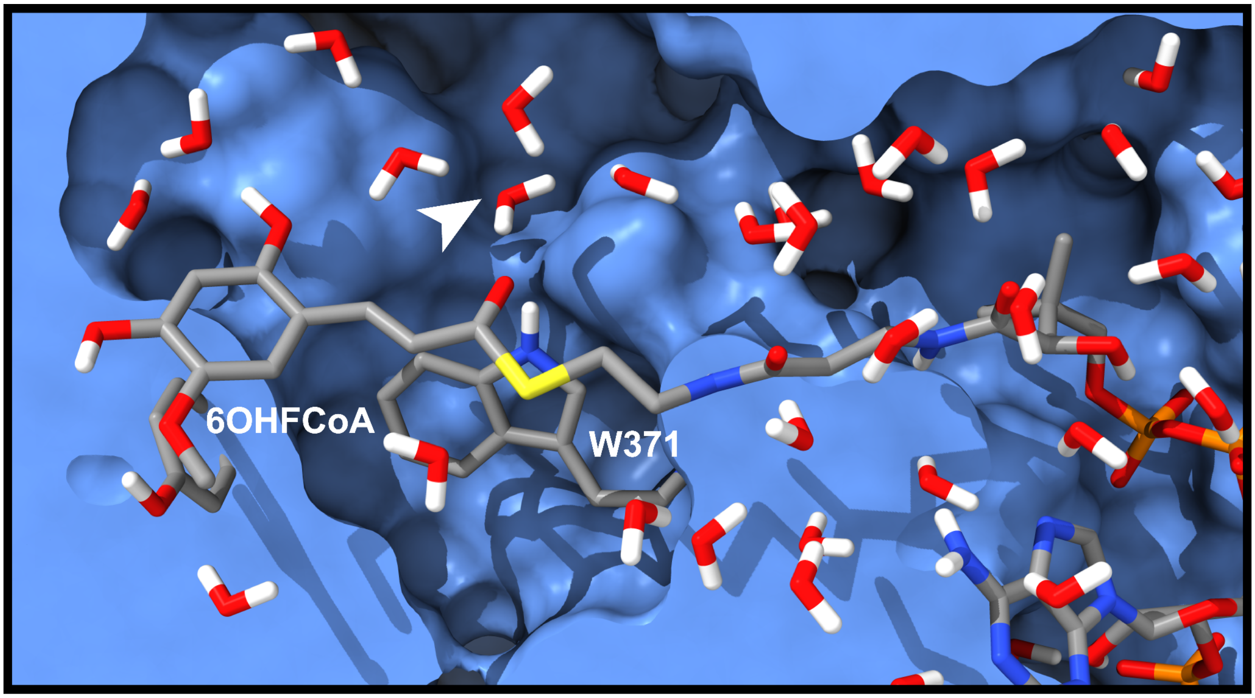

Supplementary Fig. 22 | Water configuration from MD simulations. The water configuration of the active site from the clustered MD simulation. A white arrow indicates the location of the proposed catalytic hydroxide. The substrate 6OHFCoA and Trp371 are labeled. The protein environment is highlighted as light blue. Atoms are colored as follows: carbons in gray, sulfur in yellow, nitrogen in blue, oxygen in red, and hydrogen in white.

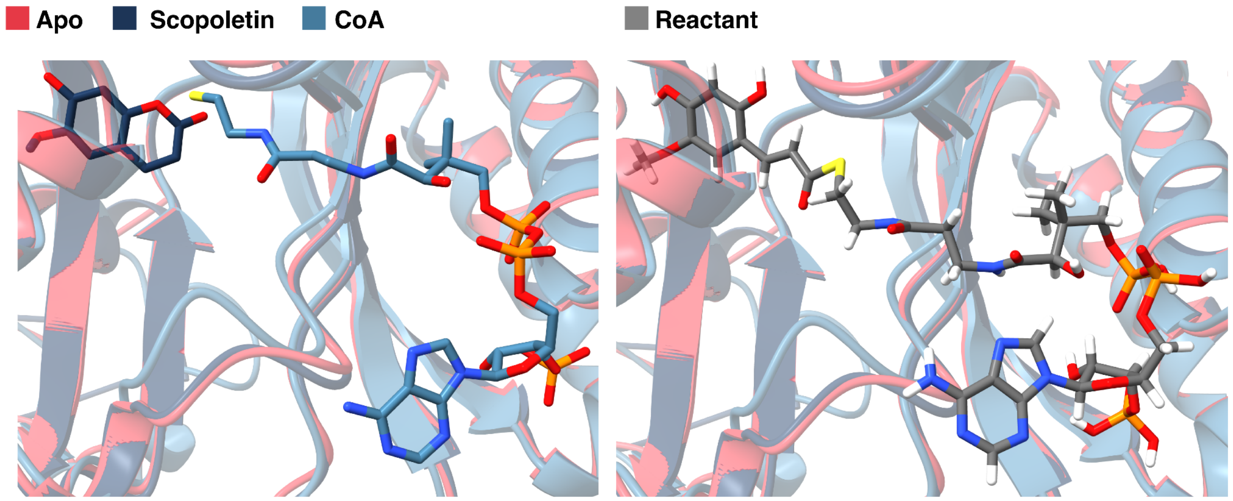

Supplementary Fig. 23 | Modeling the reactant 6-Hydroxy-feruloyl-CoA. (**left**) A depiction of the scopoletin-bound, CoA-bound, and apo crystal structures superimposed. Scopoletin is depicted with black carbons, and CoA is depicted with blue carbons. (**right**) The final 6-Hydroxy-feruloyl-CoA structure modeled by adding hydrogens and connecting scopoletin and CoA and performing a geometry optimization at the B3LYP/6-31G* level of theory. The combined ChimeraX session file is provided as a Source Data file.

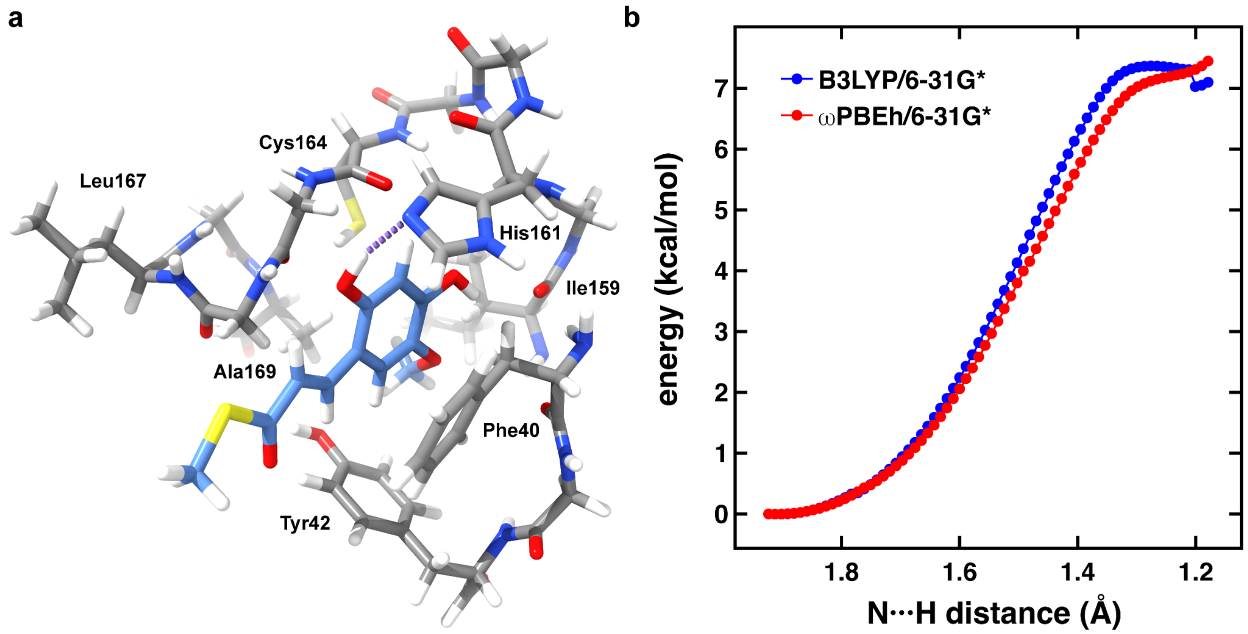

Supplementary Fig. 24 | Dissociation of a hydrogen from 6OHFCoA to His161. (**a**) The minimal cluster model used for the constrained geometry scans. Substrate carbons are colored light blue and the hydrogen bond between the substrate and His161 is shown as a dotted purple line. (**b**) Constrained geometry energy scans following the N···H coordinate between the substrate and His161. The energies were calculated with both B3LYP (blue) and wPBEh (red). Relevant dissociation scan .xyz atomic coordinate files are provided as a Source Data file.

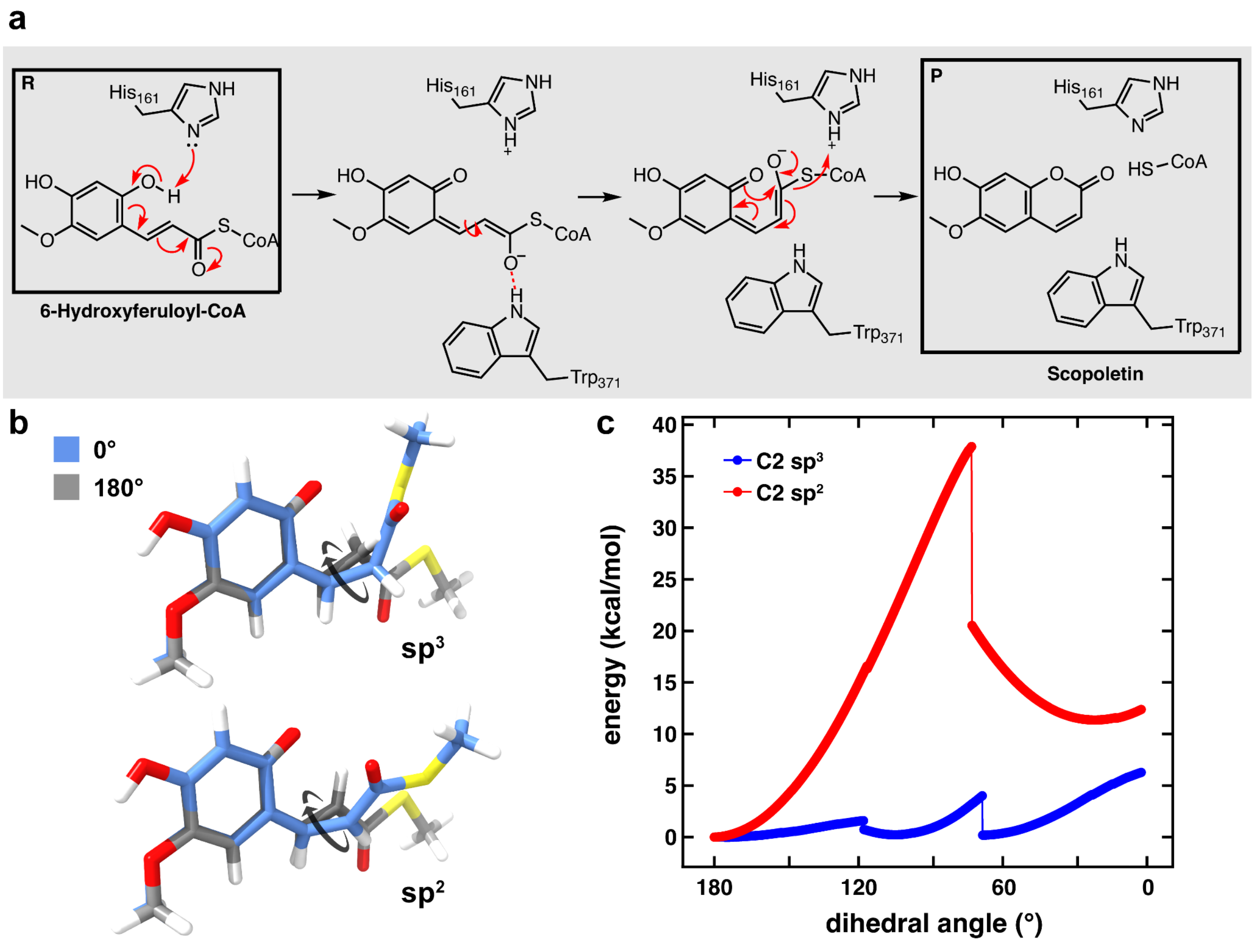

Supplementary Fig. 25 | Energetic evaluation of the *s-trans*-to-*s-cis* isomerization. (**a**) Proposed mechanism from Vanholme et al.[4] for the *trans*-to-*cis* isomerization of 6OHFCoA via intramolecular electron delocalization prior to lactonization. This mechanism does not entail proton exchange at C2. (**b**) (top) The structure of the C2-protonated substrate with the C1-C2(sp^3)-C3-C4 dihedral at 0° (gray) and 180° (blue). (bottom) The structure of the conjugated substrate with the C1-C2(sp^2)-C3-C4 dihedral at 0° (blue) and 180° (gray). (**c**) The energy profile calculated from the constrained geometry scan for the C2-protonated substrate (blue) and the conjugated substrate (red). The constrained geometry scan of the protonated species reveals two peaks at 1.6 kcal/mol and 4.0 kcal/mol. The smaller peak corresponds with a brief steric clash between one of the C2 hydrogens and the ring carbonyl. The putative transition state identified for the isomerization of the conjugated species is 37.8 kcal/mol. The drop in energy for the C1-C2(sp^2)-C3-C4 dihedral scan occurs when the hydrogen on C2 is repositioned once the double bond is finally broken. Relevant isomerization scan .xyz atomic coordinate files are provided as a Source Data file.

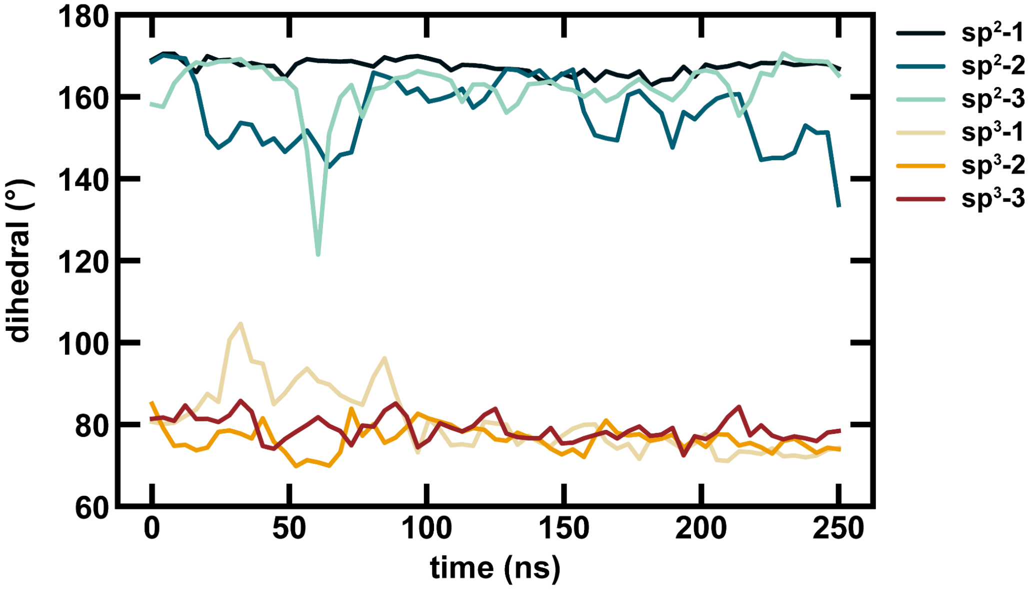

Supplementary Fig. 26 | Isomerization with either an sp^2 or sp^3 carbon at C2. 250 ns MD simulations were performed for *At*COSY with either an sp^2 (dark blue, blue, and light blue) or sp^3 carbon (red, orange, yellow) at C2 of the substrate. For each case, production dynamics were collected for three replicates at 2 ps intervals and averaged across 4 ns intervals to minimize noise. For all simulations, the substrate started with a dihedral angle of 180° for the C1-C2-C3-C4 dihedral. All replicates with a sp^3 carbon at C2 readily isomerized during the equilibration steps to a dihedral of approximately 80°, which remained relatively constant throughout the simulations. Relevant raw data from MD simulations is provided as a Source Data file.

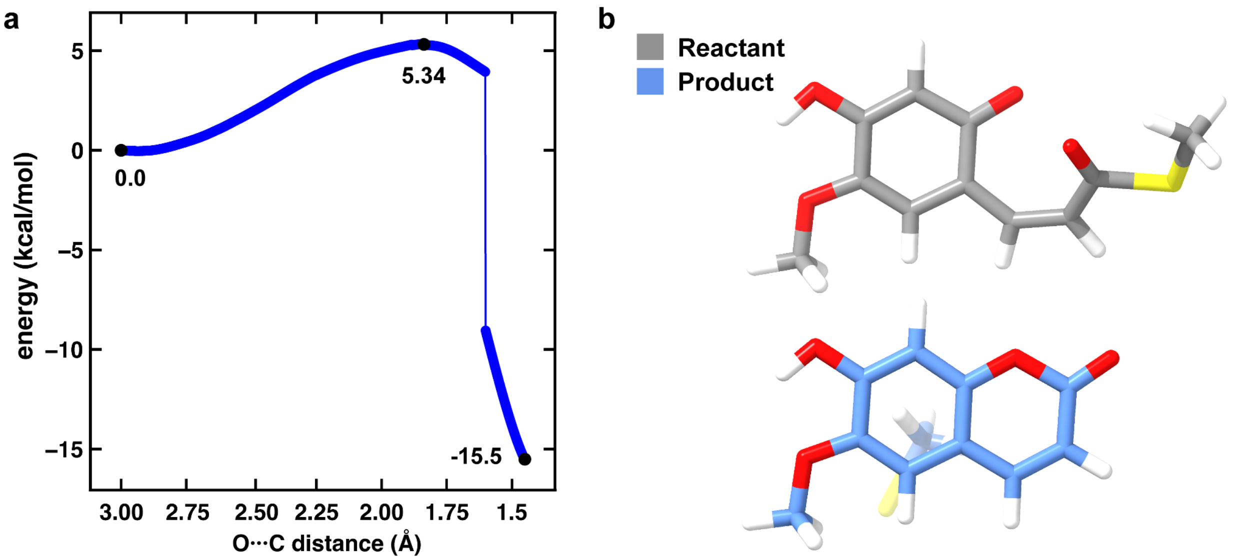

Supplementary Fig. 27 | Energetic evaluation of the lactonization step in implicit solvent with dielectric constant of water. (**a**) The energy profile calculated from the constrained geometry scan for lactonization following the trans to cis isomerization. The large drop in energy after the transition state is due to the breaking of C-S bond representative of CoA release. (**b**) (top) The structure of the starting cis conformation (gray). (bottom) The structure of the product following lactonization (blue). Relevant lactonization scan .xyz atomic coordinate files are provided as a Source Data file.

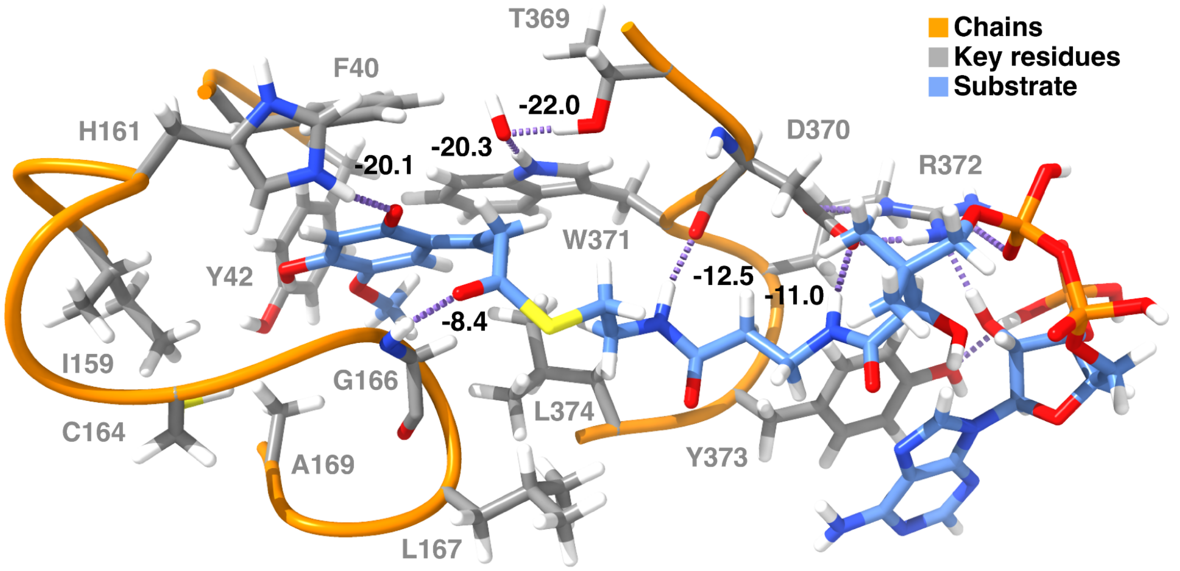

Supplementary Fig. 28 | Quantum mechanical hydrogen bonding analysis of the high energy IM3 intermediate. A hydrogen bond with Gly166, unique to the *s-cis* isomer, stabilizes the high energy conformation by -8.4 kcal/mol. Trp371 and Thr369 stabilize the hydroxide species by -20.3 and -22.0 kcal/mol respectively. Continuous chains of amino acids are shown as orange ribbons and included side chains are shown with gray carbons. The substrate is depicted with blue carbons. Hydrogen bonds are depicted as dotted purple lines. Hydrogen bond strengths are labeled in black, and residues are labeled in gray.

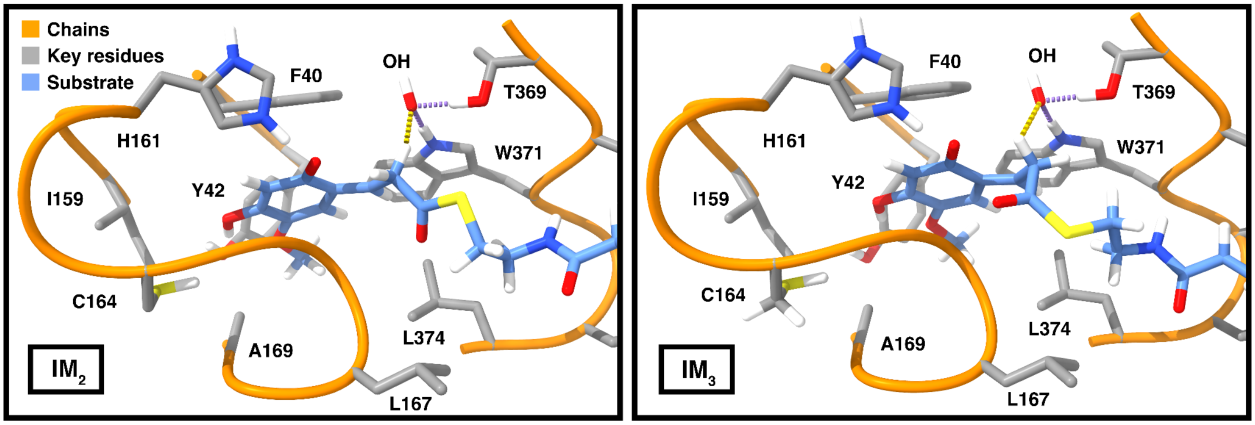

Supplementary Fig. 29 | Positioning of the catalytic hydroxide in intermediates IM_2 and IM_3. Hydrogen bonds between the proposed catalytic hydroxide and Thr369 and Trp371 are shown as purple dashed lines and the distance between the hydroxide and the target hydrogen is shown as a yellow dashed line. Continuous chains of amino acids are shown as orange ribbons and included side chains are shown with gray carbons. The substrate is depicted with blue carbons. Hydrogen bond strengths are labeled in black, and residues are labeled in gray. Relevant intermediate .xyz atomic coordinate files are provided as a Source Data file.

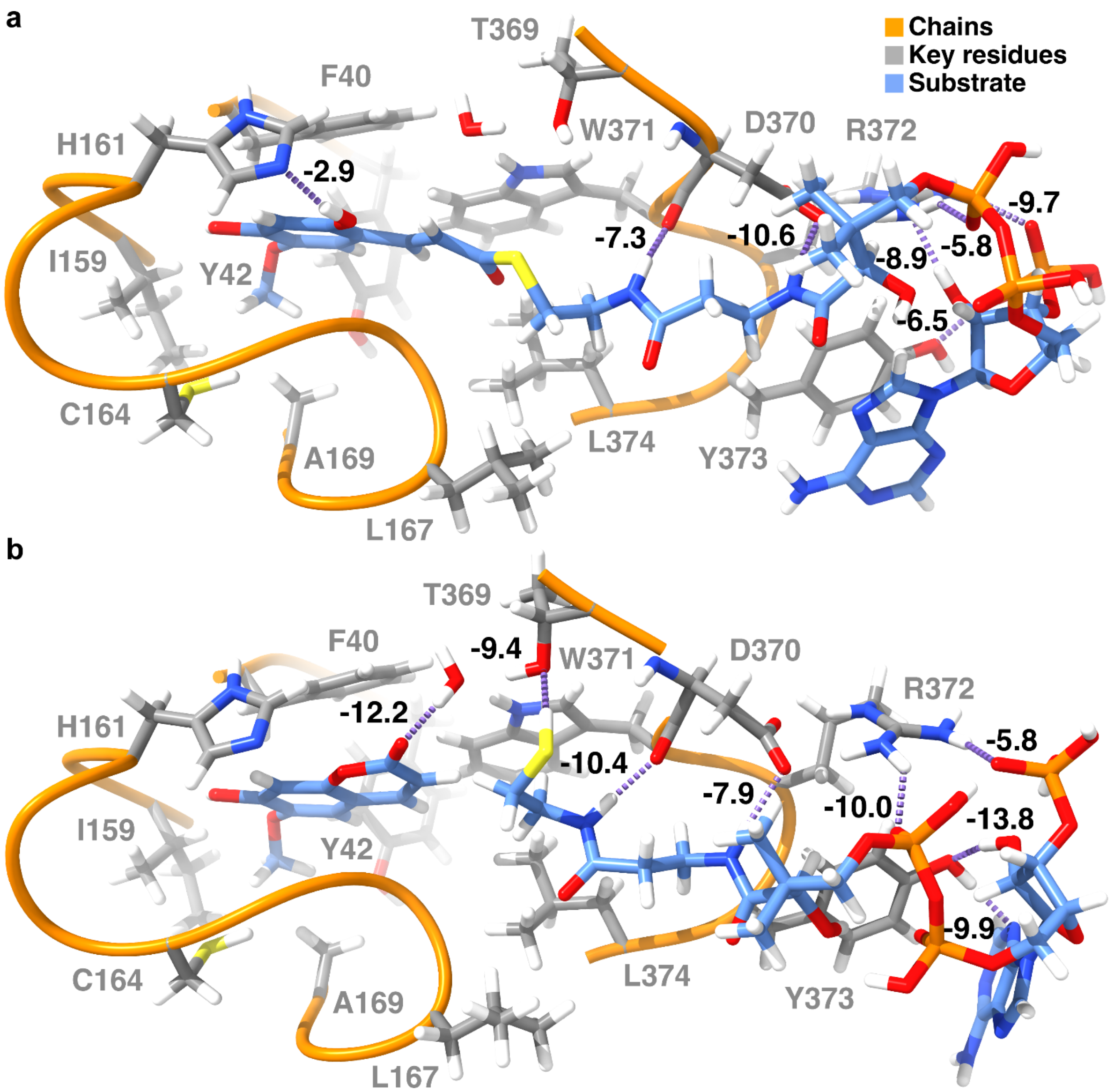

Supplementary Fig. 30 | Quantum mechanical hydrogen bonding analysis of the reactant and product states. A depiction of the hydrogen bonds between the substrate and the protein environment in the (**a**) reactant state and (**b**) product states. Hydrogen bond energies are reported in kcal/mol. Continuous chains of amino acids are shown as orange ribbons and included side chains are shown with gray carbons. The substrate is depicted with blue carbons. Hydrogen bonds are depicted as dotted purple lines. Hydrogen bond strengths are labeled in black, and residues are labeled in gray.

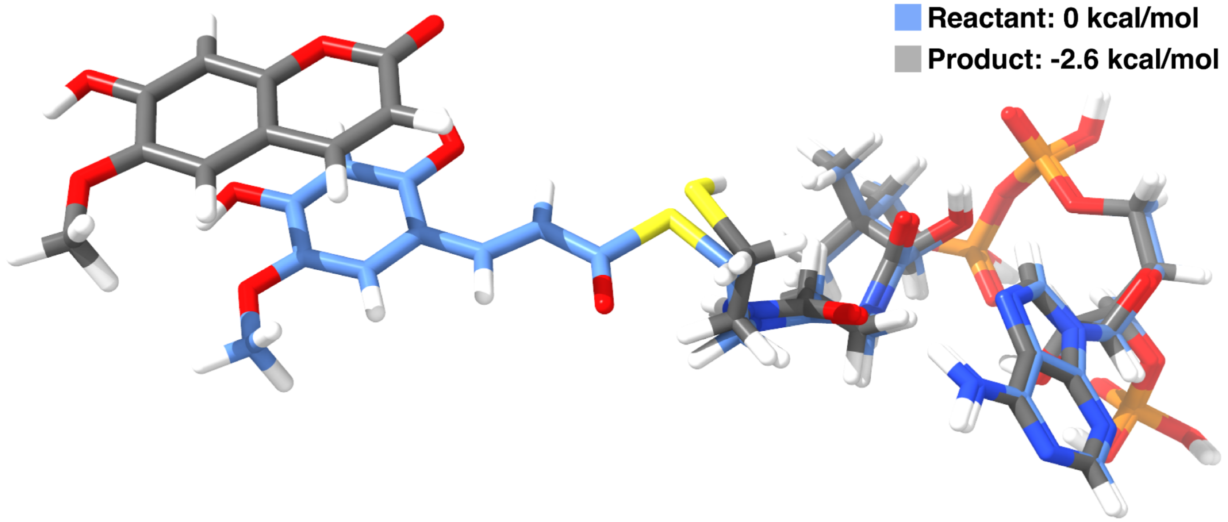

Supplementary Fig. 31 | Energetics of CoA release without the protein environment. The geometry optimized structure of the reactant (blue) and the products (grey) without the protein environment.

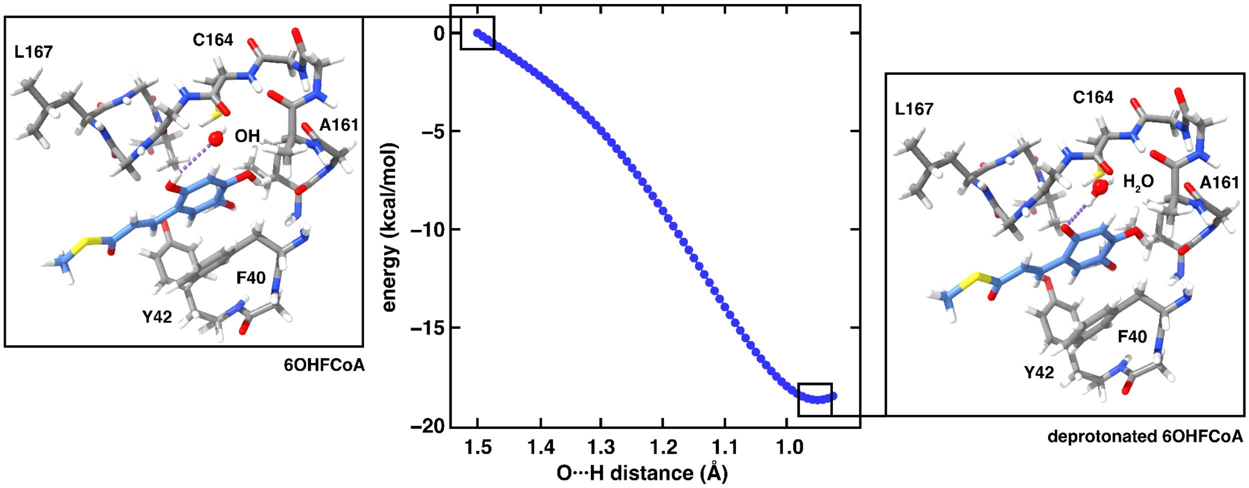

Supplementary Fig. 32 | Dissociation of a proton from 6OHFCoA to hydroxide in His161Ala mutant. A constrained geometry scan was performed using a minimal cluster model where the distance between the target hydrogen of 6OHFCoA and the oxygen of a free hydroxide was used as a reaction coordinate and is indicated with a purple dashed line. 6OHFCoA is highlighted with light blue carbons whereas the protein backbone carbons are colored in gray. Remaining atoms are colored as follows: sulfur in yellow, nitrogen in blue, oxygen in red, and hydrogen in white. Relevant hydroxide dissociation scan .xyz atomic coordinate file is provided as a Source Data file.

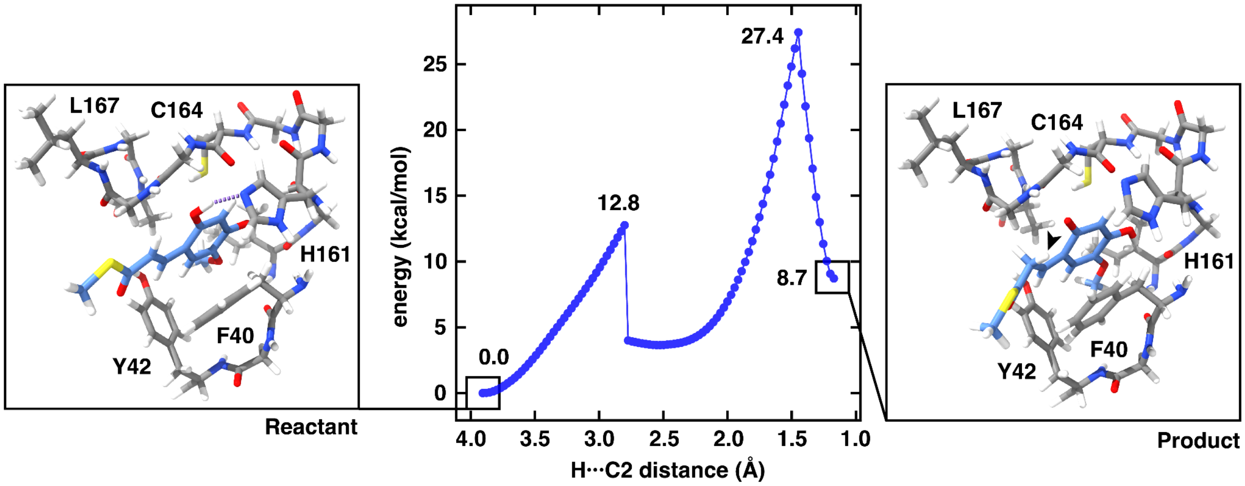

Supplementary Fig. 33 | Energetics of the intramolecular transfer of *o*-hydroxy hydrogen to C2 of 6OHFCoA. A constrained geometry scan of the alternate pathway for the protonation of C2 via the intramolecular transfer of a hydrogen atom was performed. The scan revealed two energetic barriers. The first barrier of 12.8 kcal/mol corresponds to the breaking of the strong hydrogen bond between the substrate and His161 and is shown as a purple dashed line. The second barrier of 27.4 kcal/mol corresponds to the protonation of C2. The position of the transferred hydrogen atom in the product is indicated with a black arrow. 6OHFCoA is highlighted with light blue carbons whereas the protein backbone carbons are colored in gray. Remaining atoms are colored as follows: sulfur in yellow, nitrogen in blue, oxygen in red, and hydrogen in white. Relevant intramolecular transfer scan .xyz atomic coordinate file is provided as a Source Data file.

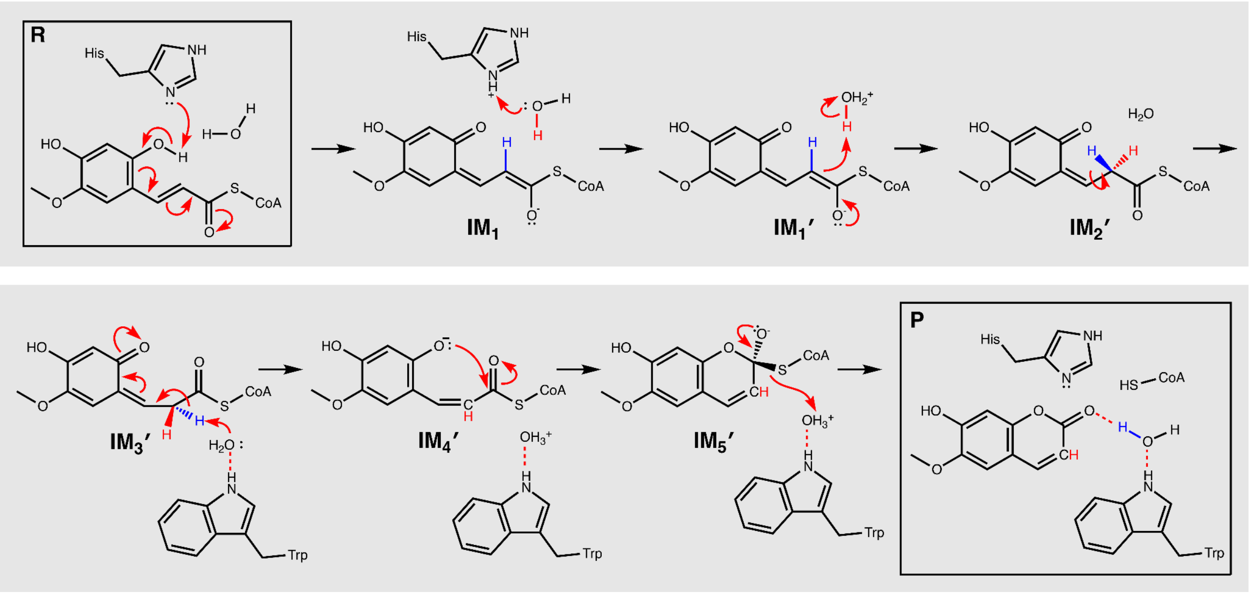

Supplementary Fig. 34 | Proposed alternative acid-base pathway. Schematic depicting an alternate acid-base mechanism that proceeds via a hydronium carrier. Stereospecific hydrogens are labeled either red or blue.

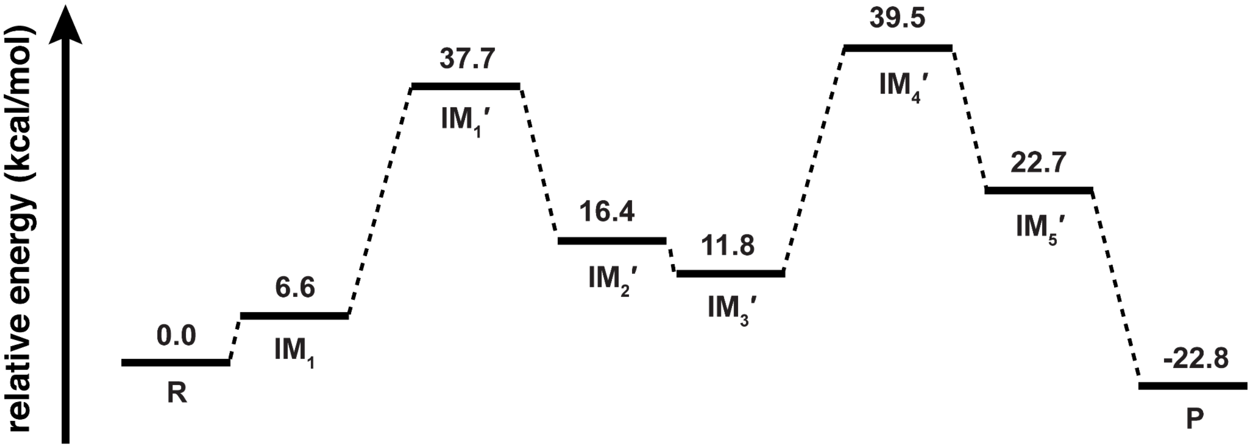

Supplementary Fig. 35 | Energetics of the alternative acid-base pathway. Energies are reported in kcal/mol. IM1′ and IM4′ are the highest energy intermediate. The highest energy intermediates contain hydronium.

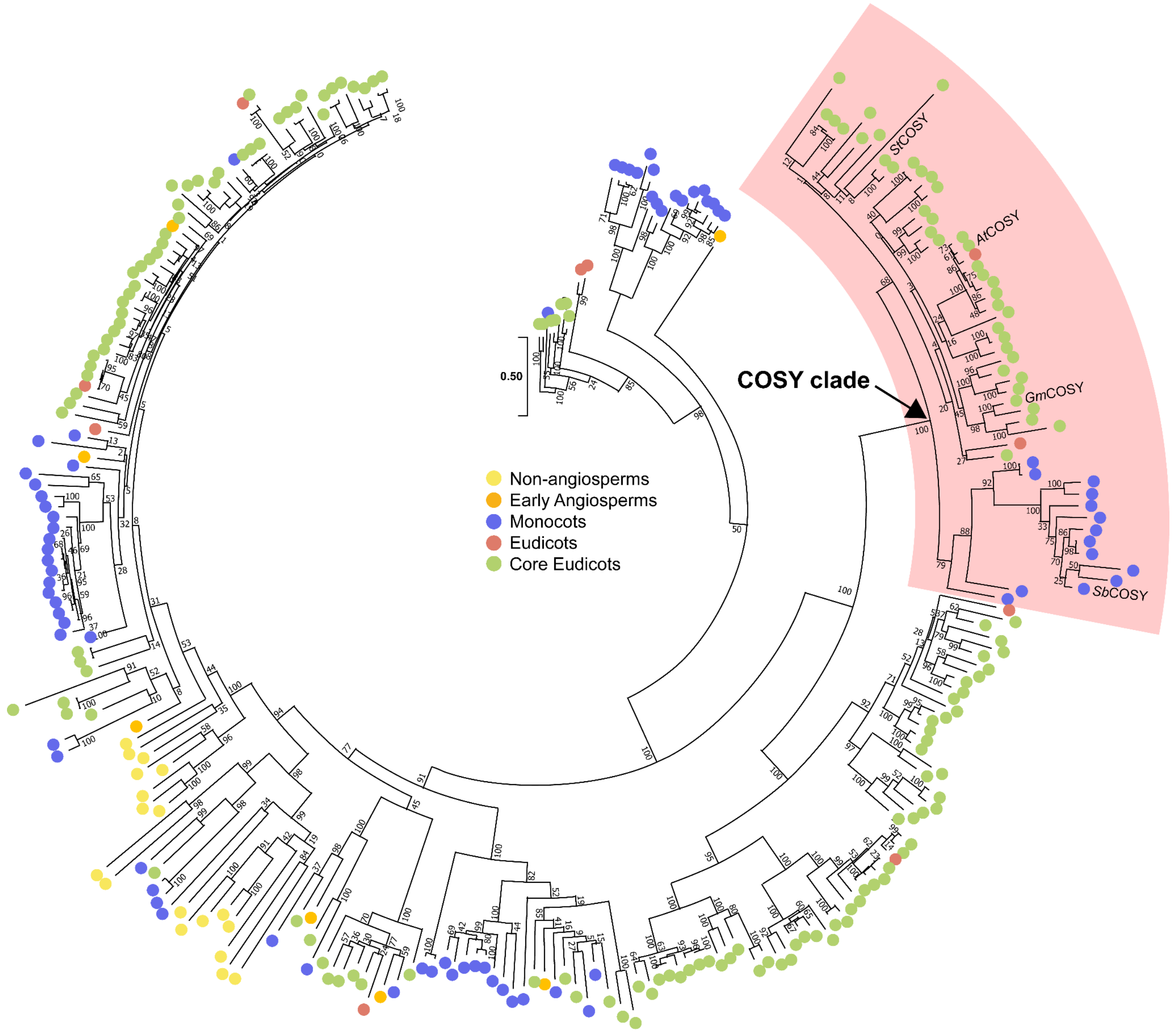

Supplementary Fig. 36 | Maximum likelihood phylogenetic tree of *At*COSY with its homologous sequences. The phylogenetic tree of COSY is built with top 5, complete sequences from pBLAST search against select species in Phytozome v.12.1.6 proteome database. Bootstrap statistics (200 replicates) are indicated at the tree nodes. The scale measures evolutionary distance in substitutions per amino acid. Sequences from species that belong to non-angiosperms, early-angiosperms, monocots, eudicots, and core eudicots are colored as yellow, orange, purple, red, and green, respectively. The arrow points to the COSY-specific clade highlighted in pink, which includes homologous sequences from monocots, eudicots, and core eudicots. *St*COSY, *At*COSY, *Gm*COSY, and *Sb*COSY are labeled at the branch tips in the COSY clade. Relevant alignment and tree files are provided as a Source Data file.

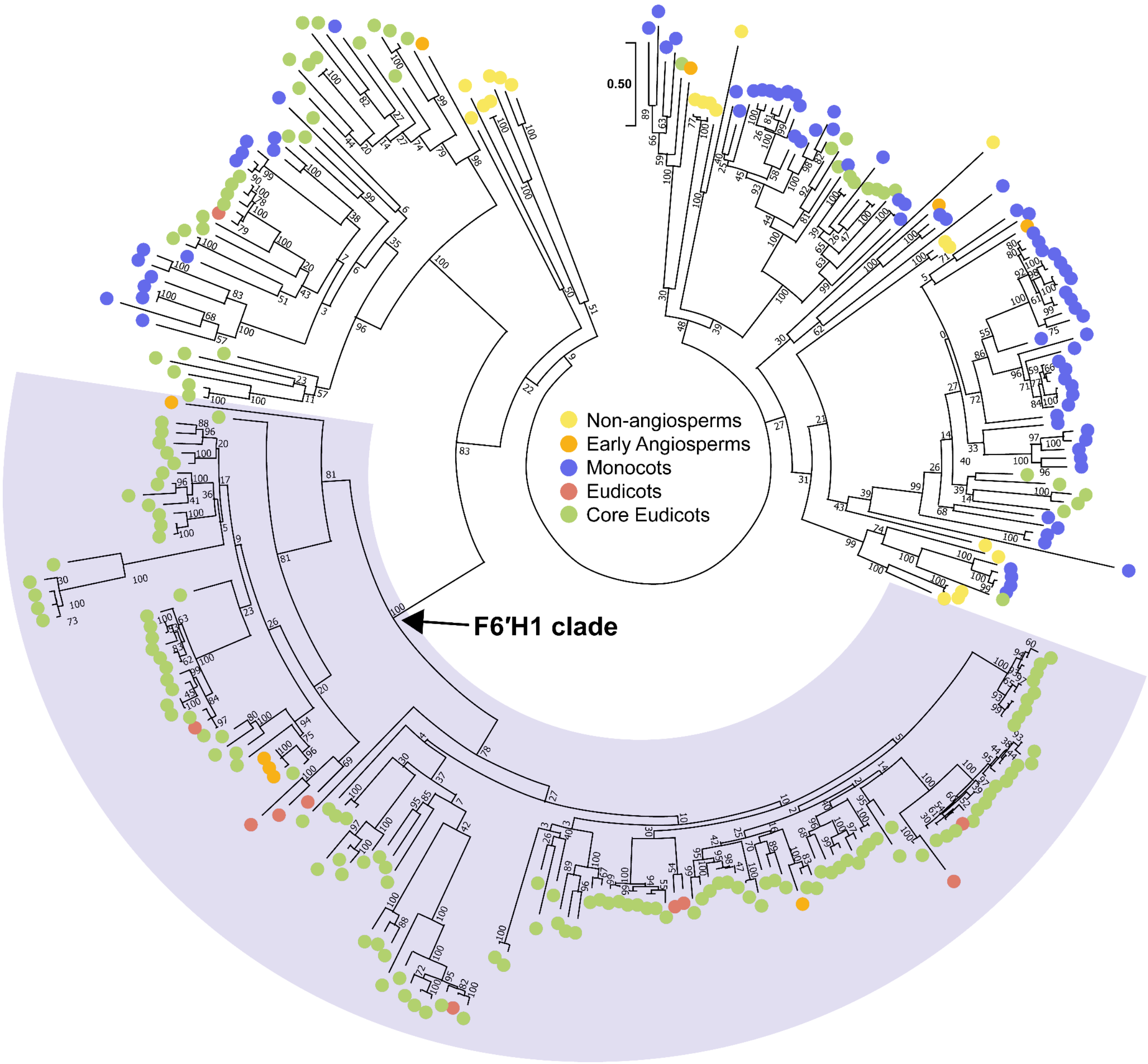

Supplementary Fig. 37 | Maximum likelihood phylogenetic tree of *At*F6′H1 with its homologous sequences. The phylogenetic tree of F6′H1 is built with top 5, complete sequences from pBLAST search against select species in Phytozome v.12.1.6 proteome database. Bootstrap statistics (200 replicates) are indicated at the tree nodes. The scale measures evolutionary distance in substitutions per amino acid. Sequences from species that belong to non-angiosperms, early-angiosperms, monocots, eudicots, and core eudicots are colored as yellow, orange, purple, red, and green, respectively. The arrow points to the F6′H1-specific clade highlighted in light-purple which includes homologous sequences from early-angiosperms, eudicots, and core eudicots. Relevant alignment and tree files are provided as a Source Data file.

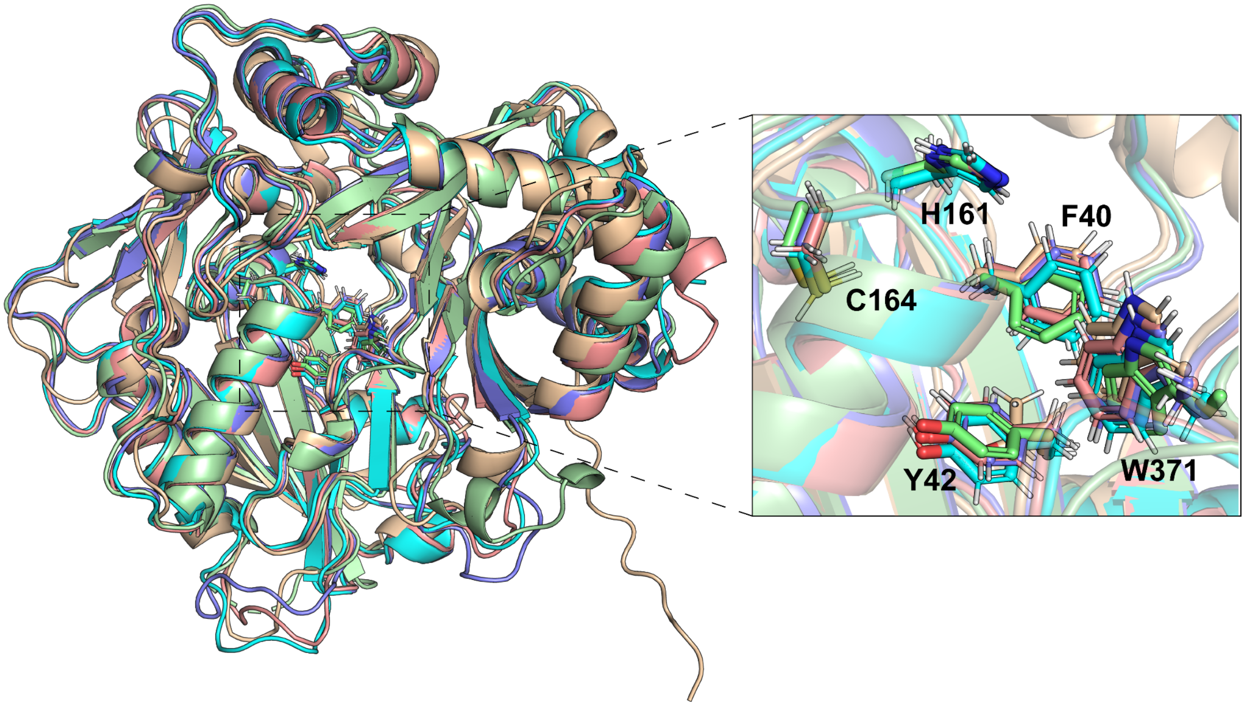

Supplementary Fig. 38 | Structural alignment of apo-*At*COSY and predicted structures of *At*COSY, *Gm*COSY, *St*COSY, and *Sb*COSY. AlphaFold2.0 was used to predict the structures. The RMSD values were calculated by the align function on PyMOL; *At*COSY (blue) = 0.992, *St*COSY (cyan) = 0.952, *Gm*COSY (pink) = 0.932, and *Sb*COSY (NCBI Accession # XM_002437812.2) (beige) = 1.389. The crystallized Apo-*At*COSY structure is portrayed in pale green. Active-site-lining residues, Phe40, Tyr42, His161, Cys164, and Trp371 numbered according to *At*COSY, exhibit similar positioning and are shown in sticks. *Sb*COSY harbors a Y42T substitution. Relevant .pdb structural models are provided as a Source Data file.

Supplementary References

1. Swift, M. L. GraphPad Prism, Data Analysis, and Scientific Graphing. *Journal of Chemical Information and Computer Sciences* vol. 37 411–412 Preprint at https://doi.org/10.1021/ci960402j (1997).
2. Edgar, R. C. MUSCLE: multiple sequence alignment with high accuracy and high throughput. *Nucleic Acids Res.* **32**, 1792–1797 (2004).
3. Robert, X. & Gouet, P. Deciphering key features in protein structures with the new ENDscript server. *Nucleic Acids Res.* **42**, W320–4 (2014).
4. Vanholme, R. *et al.* COSY catalyses trans–cis isomerization and lactonization in the biosynthesis of coumarins. *Nature Plants* vol. 5 1066–1075 Preprint at https://doi.org/10.1038/s41477-019-0510-0 (2019).
