# Supplementary material for: Emergence of a proton exchange-based isomerization and lactonization mechanism in the plant coumarin synthase COSY
Source: Nat Commun. 2023 Feb 3;14:597. doi: 10.1038/s41467-023-36299-1 (PMC9898226; doi:10.1038/s41467-023-36299-1)
Supplement: Supplementary file 3 — Reporting Summary [file 41467_2023_36299_MOESM3_ESM.pdf]

nature portfolio

Corresponding author(s): Jing-Ke Weng

Last updated by author(s): 01/07/2023

Reporting Summary

Nature Portfolio wishes to improve the reproducibility of the work that we publish. This form provides structure for consistency and transparency in reporting. For further information on Nature Portfolio policies, see our Editorial Policies and the Editorial Policy Checklist.

Statistics

For all statistical analyses, confirm that the following items are present in the figure legend, table legend, main text, or Methods section.

n/a	Confirmed	
☐	☒	The exact sample size (*n*) for each experimental group/condition, given as a discrete number and unit of measurement
☐	☒	A statement on whether measurements were taken from distinct samples or whether the same sample was measured repeatedly
☐	☒	The statistical test(s) used AND whether they are one- or two-sided<br>*Only common tests should be described solely by name; describe more complex techniques in the Methods section.*
☒	☐	A description of all covariates tested
☒	☐	A description of any assumptions or corrections, such as tests of normality and adjustment for multiple comparisons
☐	☒	A full description of the statistical parameters including central tendency (e.g. means) or other basic estimates (e.g. regression coefficient) AND variation (e.g. standard deviation) or associated estimates of uncertainty (e.g. confidence intervals)
☐	☒	For null hypothesis testing, the test statistic (e.g. *F*, *t*, *r*) with confidence intervals, effect sizes, degrees of freedom and *P* value noted<br>*Give P values as exact values whenever suitable.*
☒	☐	For Bayesian analysis, information on the choice of priors and Markov chain Monte Carlo settings
☒	☐	For hierarchical and complex designs, identification of the appropriate level for tests and full reporting of outcomes
☒	☐	Estimates of effect sizes (e.g. Cohen's *d*, Pearson's *r*), indicating how they were calculated

Our web collection on statistics for biologists contains articles on many of the points above.

Software and code

Policy information about availability of computer code

Data collection	Data collection was done using the following softwares:<br>LC-MS - Chromeleon v7.2.10 ES, TSQ Tune v3.1.279.9, XCalibur v4.5 (Thermo Fisher Scientific)<br>Sequence analysis and phylogenetic tree - MEGAX, ESPript 3, Phytozome v12.1.6, NCBI pBLAST v2.12.0<br>QM cluster model - TeraChem v1.9-2021.10-dev, Multiwfn v3.8-dev<br>Molecular dynamics - AMBER v18, H++ webserver v4.0, Gaussian v16.C.01, PMEMD v18
Data analysis	Most data analysis was done using the same softwares as data collection.<br>Protein structure analysis - PyMOL v2.4.2, PHENIX v1.19.2, Refmac v5.2, Coot v0.7.1, EMBL-HH Auto-Rickshaw, ChimeraX v1.3<br>LC-MS graphs - Xcalibur Qual Browser v4.5 (Thermo Fisher Scientific)<br>Bar graphs, scatter plots, other quantitative graphs - GraphPad Prism (v. 9.0)<br>Figure compilation - Adobe Illustrator v26.3.1.

For manuscripts utilizing custom algorithms or software that are central to the research but not yet described in published literature, software must be made available to editors and reviewers. We strongly encourage code deposition in a community repository (e.g. GitHub). See the Nature Portfolio guidelines for submitting code & software for further information.

Data

Policy information about availability of data

All manuscripts must include a data availability statement. This statement should provide the following information, where applicable:

- Accession codes, unique identifiers, or web links for publicly available datasets
- A description of any restrictions on data availability
- For clinical datasets or third party data, please ensure that the statement adheres to our policy

All atomic coordinates and structure factors generated in this study have been deposited in the Protein Data Bank database under accession codes 8DQO [https://doi.org/10.2210/pdb8DQO/pdb] (COSY Apo), 8DQP [https://doi.org/10.2210/pdb8DQP/pdb] (COSY + scopoletin), 8DQQ [https://doi.org/10.2210/pdb8DQQ/pdb] (COSY + umbelliferone), and 8DQR [https://doi.org/10.2210/pdb8DQR/pdb] (COSY + CoA). The native HCT structure from C. canephora used for molecular replacement was obtained from RCSB Protein Data Bank under identifier 4G0B. Relevant LC-HRAM-MS raw data files generated in this study have been deposited in Zenodo [https://doi.org/10.5281/zenodo.7513211]. The genomic sequences used for phylogenetic tree construction can be obtained in Phytozome v12.1.6 database and data underlying Supplementary Figures 36 and 37 are provided in the Source Data file. AlphaFold2.0 structural models generated for Supplementary Figure 38 are also provided in the Source Data file. Atomic coordinate files used in computational analyses are included in the Source Data file. Data is also available from the corresponding author upon request.

Human research participants

Policy information about studies involving human research participants and Sex and Gender in Research.

Reporting on sex and gender	Reporting on sex and gender was not relevant to our study.
Population characteristics	Population characteristics were not relevant to our study.
Recruitment	Recruitment was not relevant to our study.
Ethics oversight	Ethics oversight was not relevant to our study.

Note that full information on the approval of the study protocol must also be provided in the manuscript.

Field-specific reporting

Please select the one below that is the best fit for your research. If you are not sure, read the appropriate sections before making your selection.

[x] Life sciences [ ] Behavioural & social sciences [ ] Ecological, evolutionary & environmental sciences

For a reference copy of the document with all sections, see nature.com/documents/nr-reporting-summary-flat.pdf

Life sciences study design

All studies must disclose on these points even when the disclosure is negative.

Sample size	All qualitative traces of LC-HRAM-MS data reported in this study are displayed as n = 1, though duplicate or triplicate data have been recorded (only one trace per sample is shown because they are redundant). Quantitative data from LC-HRAM-MS experiments are noted as n = 2 or n =3 (as described in each associated Figure caption). Sample size was determined based on the consistency of the enzyme assay results, which was steady with duplicate or triplicate samples. Unpaired, two-tailed t-test was performed for statistical comparison between samples using GraphPad Prism v9.0.<br><br>For the in planta deuterium-feeding study of WT and cosy Arabidopsis mutant, 20 root tissues were grown for 20 days on 1/2 MS solid media and pooled for analysis.
Data exclusions	No data exclusions apply in our study.
Replication	For COSY in vitro enzyme assays, 2-3 biological replicates were used as depicted in main text, methods, or figure captions. For the in planta deuterium-feeding study of WT and cosy Arabidopsis mutant, 3 technical replicates were used as mentioned in the figure caption.<br>All experiments could be replicated and results from different replicates were consistent.
Randomization	This is not relevant to our study, as samples were always analyzed by LC-MS in a random manner.
Blinding	Blinding was not plausible due to various in vitro experiments and in planta experiments that required proper identification in this study.

Reporting for specific materials, systems and methods

We require information from authors about some types of materials, experimental systems and methods used in many studies. Here, indicate whether each material, system or method listed is relevant to your study. If you are not sure if a list item applies to your research, read the appropriate section before selecting a response.

Materials & experimental systems

n/a	Involved in the study
☒	☐ Antibodies
☒	☐ Eukaryotic cell lines
☒	☐ Palaeontology and archaeology
☒	☐ Animals and other organisms
☒	☐ Clinical data
☒	☐ Dual use research of concern

Methods

n/a	Involved in the study
☒	☐ ChIP-seq
☒	☐ Flow cytometry
☒	☐ MRI-based neuroimaging
